# Feasibility, reproducibility and validity of the 10 meter Shuttle Test in mild to moderately impaired people with stroke

**DOI:** 10.1371/journal.pone.0239203

**Published:** 2020-10-28

**Authors:** Harriet Wittink, Tim Blatter, Jacqueline Outermans, Mariella Volkers, Paul Westers, Olaf Verschuren

**Affiliations:** 1 Research Group Lifestyle and Health, Utrecht University of Applied Sciences, Utrecht, The Netherlands; 2 UMC Utrecht Brain Center and Center of Excellence for Rehabilitation Medicine, Utrecht University, Utrecht, The Netherlands; 3 Julius Centre for Health Sciences and Primary Care, University Medical Center Utrecht, Utrecht, The Netherlands; Newcastle University, AUSTRALIA

## Abstract

**Background:**

There currently is no field test available for measuring maximal exercise capacity in people with stroke.

**Objective:**

To determine the feasibility, reproducibility and validity of the Shuttle Test (ST) to measure exercise capacity in people with stroke.

**Design:**

Longitudinal study design.

**Setting:**

Rehabilitation department, day care centres from a nursing home and private practices specialized in neuro rehabilitation.

**Subjects:**

People with subacute or chronic stroke.

**Interventions:**

A standardized protocol was used to determine feasibility, reproducibility and validity of the 10-meter Shuttle Test (10mST).

**Main measures:**

Number of shuttles completed, 1^st^Ventilatory Threshold (1^st^VT).

**Results:**

The associations of the number of shuttles completed and cardiopulmonary capacity as measured with a portable gas analyser were r > 0.7, confirming good convergent validity in subacute and chronic people with stroke. Criterion validity, however, indicates it is not a valid test for measuring maximal cardiopulmonary capacity (VO_2max_). Only 60% of participants were able to reach the 1^st^VT. Higher cardiopulmonary capacity and a higher total score of the lower extremity Motricity Index contributed significantly to a higher number of shuttles walked (p = 0.001).

**Conclusions:**

The Shuttle Test may be a safe and useful exercise test for people after stroke, but may not be appropriate for use with people who walk slower than 2 km/h or 0.56 m/s.

## Introduction

Rehabilitation of people with stroke is aimed at gaining as much independence as possible in their own environment. Gaining or maintaining adequate cardiopulmonary capacity is an essential requirement for maintaining physical function [1].

The assessment of maximal cardiopulmonary capacity in people with stroke is more challenging than in healthy subjects because they present with stroke-specific impairments such as muscle weakness, fatigue, poor balance, contractures and spasticity, which can compromise maximal cardiopulmonary exercise test outcome [2, 3]. Given the reported difficulty of measuring VO_2max_ in people with stroke [4], we may need to consider alternatives, for instance, the determination of the first ventilatory threshold (1^st^VT). The 1^st^VT represents the intensity limit of prolonged exercise above which a transition to anaerobic metabolism begins [5]. As the intensity of exercise begins to increase, the 1^st^VT can be identified at the point where the breathing rate begins to increase. It is recommended as an appropriate target intensity level for the prescription of light to moderate exercise. It has been suggested the 1^st^VT may be a more specific measure of cardiopulmonary capacity than peak oxygen uptake in people with stroke [5].

Apart from the challenges of maximal cardiopulmonary exercise testing in people with stroke, it requires expensive equipment and trained personnel, not available to many clinicians. Consequently, there is an urgent need for a valid and reproducible field test to assess at least the 1^st^VT and if possible, VO_2max_. In clinical settings field tests of walking ability such as the six-minute walk test (6MWT) are often used. Prior studies have concluded that the 6MWT is not an adequate measure of cardiopulmonary capacity after stroke [6–9], as the number meters walked in the 6MWT only has a moderate correlation with VO_2peak_ as measured in a maximal symptom limited exercise test [10].

The 10 meter Shuttle Test (10mST), as modified for people with Cerebral Palsy [11], may be of more use. For many adults with physical disabilities, the original 20-m shuttle test is not suitable, because the starting speed (8 km/h) and increase (0.5 km/h) every minute are beyond their capabilities. The adapted protocol, with a slower starting speed (2.0 km/h) and smaller speed increases (0.25 km/h), has been found to be safe and to have acceptable reproducibility and convergent validity for children with Cerebral Palsy [11]. In this protocol, the number of shuttles is the primary outcome, which has been found to be reproducible with an ICC_2,1_ = 0.99 [11] in children with cerebral palsy.

High test-retest reliability (ICC_2,1_ = 0.96) with a standard error of measurement of 6% and high significant correlations (*r*_p_ = 0.65, p<0.01) between the 10mST and 6MWT have been found in people with chronic stroke [12]. No studies have investigated the ability of the 10mST to accurately assess cardiopulmonary capacity in people with subacute or chronic stroke. Results may be different in people with subacute stroke compared with people with chronic stroke, as the process of motor recovery may confound reproducibility results.

The primary objective of this study was to investigate the feasibility, reproducibility, criterion and convergent validity of the 10mST to measure cardiopulmonary capacity in people with stroke.

Our secondary objective was to determine if there are differences in the feasibility, reproducibility, criterion and convergent validity of the 10mST during the subacute versus the chronic phase after stroke.

## Methods

### Recruitment and subjects

People with stroke receiving rehabilitation were recruited from March–December 2017 from a rehabilitation centre (subacute participants), or from day therapy centres within residential care facilities or private practices specialized in neuro rehabilitation (chronic participants) in Utrecht, the Netherlands. Subjects were included in the present study if they had a diagnosis of stroke according to the WHO definition [13], were aged over 18 years, able to walk with supervision (Functional Ambulation Categories ≥ 3) and be in the final week prior to discharge from the rehabilitation centre. Subacute stroke was defined as time since stroke between 7 days and 6 months and chronic stroke was defined as time since onset > 6 months [14].

Subjects were excluded if they presented with severe cognitive disorder (Mini Mental State Examination <24 points), severe communicative disorder (Utrecht Communication State < 4 points), if they had contraindications to maximal exercise [15], such as severe cardiovascular disease or if they were a recurrent faller, defined as more than two falls in a six‐month period [16]. Falls were defined as “an unexpected event in which the participants come to rest on the ground, floor, or lower level” [17].

The study was approved by the Medical Ethical Board of the Utrecht Medical Center. Written informed consent was obtained from each subject. Subjects were treated in accordance to good clinical practice and the declaration of Helsinki [18].

### Procedure

Data were collected by two experienced exercise physiologists (TB and MV). Both assessors were Automated External Defibrillator (AED)-trained. A physician was always stand-by in the immediate surroundings to act in case of emergency. Prior to the first assessment subjects were asked to perform only light physical activity on the day before testing, and not to exercise or smoke for 2 hours before their test visits as it is known this can influence cardiopulmonary capacity test results.

At the first test visit, pre-participation health screening using the American Heart Association /American College of Sports Medicine Health/Fitness Pre-participation Screening Questionnaire [19] was performed according to guidelines of the American College of Sports Medicine [19]. Following the screening, demographic data were collected on age, sex, lesion type, side of stroke and side of hemiplegia Height and weight were measured using calibrated measurement instruments at the first and second visit, on average 1 week later. Recurrent falls were defined based on self-report [20]. We measured balance with the Tinetti Performance Oriented Mobility Assessment-Balance (POMA-B) [21]. For the POMA-B the minimum score is 0 and the maximum score 16 (no balance problems). Functional lower extremity strength was measured with the lower extremity Motricity Index [22]. The minimum score is 0 and the maximum score is 100 (normal strength).

The 10mST was conducted according to the protocol by Verschuren et al. [11] which starts at 2 km/h and increases speed by 0.25 km/h every level (one minute) to a maximum speed of 7.5 km/h (23 stages).

Two cones were set to provide a between-cone distance of 10 meters. The 10mST required participants to walk between the 2 markers delineating the course, at a set incremental speed determined by an auditory signal (beep), which was played by a standard CD/MP3 player. Each stage lasted about 1 minute. Participants were instructed to keep walking as long as possible, not to talk during the test and to wait to start walking until the beep had sounded. To ensure that the subjects were safe during the test, a researcher assisted them by walking behind or next to them. No gait belts or other devices were used for support, but patients were allowed to use their own assistive devices for walking. An emergency button was always within reach of the subjects and the therapist.

During the test, subjects wore a mobile gas analysis system and a portable wireless ECG (Custocor Custo Med, Ottobrunn, Germany). The mobile gas analysis system consisted of a facemask, a transmitting unit (containing different oxygen and carbon dioxide gas analysers), and a receiving unit. The transmitting unit with facemask and tubing was attached to the subjects with a harness, and the receiving unit was connected to a laptop computer located within 500 m of the transmitting unit. Metabolic stress test software (Metasoft, Version 5.4) was used to measure ECG, heart rate, breath-by-breath minute ventilation, oxygen consumption (VO_2_) carbon dioxide production (VCO_2_), and to calculate the Respiratory Exchange Ratio (RER = VCO_2_/VO_2_). The gas analysis system was fully calibrated immediately before each test as recommend by the manufacturer. The validity and reliability of this system is acceptable [23, 24].

The test finished when the subject was limited by dyspnoea or when the subject was unable to maintain the required speed and failed twice to complete a shuttle before the beep sounded. Subjects were asked not to talk during the test, but to use a hand signal to indicate they wanted to stop the test. After the test subjects were asked to walk back and forth slowly for a few minutes as a cool-down period. For perceived exertion the Borg score was measured (range 6–20) immediately after completing the 10mST test.

Subjects were then asked to rest for 20 minutes prior to engaging in a supramaximal constant work rate validation procedure bout of exercise [25] to confirm subjects had reached maximal effort and the highest shuttle they could achieve during the test. Supramaximal means a workload above the peak workload attained during the 10mST. This validation procedure consisted of a constant work rate test of maximal 3 minutes [26] at one shuttle higher than the highest shuttle reached in the 10mST.

### Outcome measures

The primary variable was the number of shuttles completed.

The secondary variables were:

1. VO_2max_ (ml.kg^-1^.min^-1^), defined as a plateau in VO_2_ or a Respiratory Exchange Rate (RER) of ≥1.05 for 50–64 year olds and RER ≥1.0 for those 65 years and older for both males and females as recommended by Edvardsen et al. [27]. We considered VO_2peak_ as the average of the last 30 sec of the 10mST.
2. 1^st^VT was determined by two independent raters using the ventilatory equivalents method, and the nadir of fraction of oxygen (P_ET_O2) according to Binder et al. [28] When the outcomes were uncertain, the V-slope method [28] was used to verify the 1^st^VT. We calculated two-way random Intraclass Correlation Coefficients with absolute agreement for the interrater reliability of 1^st^ VT and found it to be ICC_2,1_ = 0.91 with a 95% CI .38 – .98.

#### Data analyses

All analyses were performed with the statistical software package SPSS version 24 for Windows (IBM SPSS Statistics 24). All data were checked with tests of normality using Shapiro Wilks statistics. Percentages were calculated for ordinal data. When normally distributed, means and standard deviations (SD) were calculated for descriptive measures. Medians and ranges were reported for non-normally distributed data. We calculated differences in age, POMA and lower extremity Motricity Index between those who were unable to complete the 10mST (defined as inability to complete the first shuttle in the first test) and those who completed the test using independent samples t-tests or Mann-Whitney U tests as appropriate.

Linear mixed modelling was used to determine whether there were significant differences in number of completed shuttles when correcting for time of testing (test or retest), lower extremity Motricity Index, POMA-B, sex and age as the independent variables. We assumed a random intercept.

Feasibility was assessed by recording complications, such as dizziness, cardiac arrhythmias and/or adverse effects (falls) during or after the ST. In addition, we determined if subjects reached maximal effort during the ST by using a supramaximal constant work rate test.

Reproducibility concerns the degree to which repeated measurements in stable persons (test-retest) provide similar answers. In accordance with Terwee et al. [29] we made a distinction between reliability and agreement.

For reliability we calculated a two way mixed Intraclass Correlation Coefficient (ICC_2,1_) with absolute agreement of interrater reliability for the determination of the 1^st^VT, the absolute and relative VO_2peak_, HR_peak_ and RER_peak,_ distance walked and number of shuttles for the whole sample, chronic survivors and the subacute survivors [30]. For the interpretation of results, single measure ICC values less than 0.5 are indicative of poor reliability, values between 0.5 and 0.75 indicate moderate reliability, values between 0.75 and 0.9 indicate good reliability, and values greater than 0.90 indicate excellent reliability [30].

To determine the level of agreement, Standard Error of Measurement (SEM) and the smallest detectable change (SDC_ind_) were calculated according to the recommendations of Terwee et al. [29].

For cardiopulmonary criterion validity we determined if the 10mST stresses the cardiopulmonary system sufficiently to attain at least the 1^st^VT, or VO_2max_ (see definition above). We considered the test valid for stressing the cardiopulmonary system if the 1^st^VT could be determined in 80% of subjects during the 10mST.

For convergent validity we hypothesized that there would be a strong positive correlation (r ≥ 0.75) between the variables number of shuttles completed and aerobic capacity, measured as VO_2peak_ mL.min^-1^ and VO_2peak_ mL.kg^-1^.min^-1^. Correlations ranging from 0.00–0.25 are considered to indicate no relationship, those from 0.25–0.50 a fair degree of relationship, values of 0.50–0.75 a moderate to good and values above 0.75 a good to excellent relationship [30]. In addition, we performed a forward linear regression analyses with the number of shuttles completed as the dependent variable and the lower extremity Motricity Index score and VO_2peak_ mL.kg^-1^.min^-1^ as the independent variables.

Finally, we analysed whether validity and reproducibility of the 10mST was different between participants in the subacute versus the chronic phase after stroke by using unpaired t-tests and Mann-Whitney U tests as appropriate.

## Results

### Participants

Twenty-six participants were recruited for the study. No potential participants were excluded on the basis of the pre-participation screening. N = 6 (23%) were unable to complete the first shuttle in the first test. Participants who were unable to complete the first shuttle were on average older (mean difference (Standard Error (SE)) 13.6 (6.9) years, 95% CI -0.6; 27.7), had a lower POMA score mean difference (SE) 4.1 (0.7), 95% CI -5.5; -2.7) and a lower lower extremity Motricity Index (mean difference (SE) 28.3 (8.1), 95% CI -45.1; -11.5). These six participants were excluded from the retest.

Twenty participants completed the test twice. Demographic data for the test-retest sample are shown in Table 1.

**Table 1:** Demographic variables of the test-retest participants (n = 20).

	Total (n = 20)	Subacute (n = 7)	Chronic (n = 13)	p-value
Male (n (%))	9 (45)	4 (57)	5 (39)	0.42***
Age (Years, mean(SD))	60 (16)	46 (15)	67 (12)	0.005*
Height (cm, mean(SD))	171 (11)	170 (10)	171 (11)	0.87*
Weight (kg, mean(SD))	78.1 (17.1)	69.4 (15.0)	82.7 (16.9)	0.10*
Duration stroke (months, mean (SD))	62.3 (78.0)	7.2 (2.0)	87.8 (83.1)	0.004*
Hemiplegic side (n (%))				0.62***
Left	10 (50)	3 (43)	7 (53)	
Right	6 (30)	3 (43)	3 (21)	
Both	1 (5)		1 (8)	
None	1 (5)		1 (8)	
Missing	2 (10)	1 (14)	1 (8)	
Type of CVA (n (%))				0.34***
Ischaemic	14 (70)	4 (57)	10 (77)	
Haemorrhagic	5 (25)	3 (43)	2 (15)	
Unknown	1 (5)		1 (8)	
FAC (n (%))				0.48***
FAC 4	4 (20)	2 (29)	2 (15)	
FAC 5	16 (80)	5 (71)	11 (85)	
Motricity Index lower extremity (median (range))	91 (44–100)	100 (89–100)	85.0 (44–100)	0.006**
POMA (median (range))	16 (13–16)	16 (14–16)	16 (13–16)	0.94**
Beta-blockers (n (%))	3 (15)	2 (29)	1 (8)	0.33***
Assistive devices indoors (n (%))				0.30***
None	18 (90)	6 (86)	12 (92)	
Cane	1 (5)	1 (14)		
Walker / cane	1 (5)		1 (8)	
Assistive devices outdoors (n (%))				0.58***
None	15 (75)	6 (87)	9 (69)	
Cane	2 (10)	1 (14)	1 (8)	
(rolling) walker	2 (10)		2 (16)	
Walker/ cane	1 (5)		1 (8)	

*Independent t-test,

**Mann-Whitney U test,

***Chi square.

### Test retest reliability

Linear mixed modelling analysis of the whole sample with the number of completed shuttles as the dependent variables adjusting for sex, age, POMA and lower extremity Motricity Index as the dependent variables showed no significant effect of time of testing (p = 0.06). See Table 2.

**Table 2:** Test-retest reliability of the number of shuttles completed.

Variable	Estimate	t	Sig.	95% Confidence Interval	
				Lower Bound	Upper Bound
Intercept	-17.69	-1.18	0.26	-49.71	14.34
Time of testing	0.48	1.99	0.06	-0.03	0.98
Sex	3.44	1.90	0.08	-0.41	7.29
POMA	1.66	1.84	0.09	-0.26	3.58
lower extremity Motricity Index	0.110	1.80	0.09	-0.02	0.23
Age	-0.15	-2.63	0.02	-0.27	-0.03

### Feasibility

There were no adverse events during or after the 10mST, including dizziness, syncope, fainting, chest pains, cardiac arrhythmias or (near) falls while performing the 10mST. Most (n = 17 (85%)) of the participants stopped due to motor control and/or coordination problems that resulted in the inability to walk the required speed. Three (15%) participants reported stopping because they were out of breath. Median (range) Borg scores were 15 (7–17) and 16.5 (7–18) at the test and retest, respectively. The test was viable in all settings (private practice, rehabilitation and day care).

Mean (SD) minutes walked in the supramaximal test was 1.2 (0.6) minutes. No participants were able to complete the 3 minute supramaximal test, suggesting they had reached their maximal capacity during the ST.

### Reproducibility

There were no statistically significant differences between the test and retest for all measures reported in Table 3.

**Table 3:** Reproducibility Shuttle Test whole sample.

Mean (SD)	Test (n = 20)	Retest (n = 20)	Single measure ICC_1,2_ agreement 95% CI	SEM	SDC_ind_	SDC_group_
VO_2peak_ (L.min^_1^)	1.67 (0.7)	1.6 (0.7)	0.9 (0.8; 1.0)	0.2	0.5	0.1
VO_2peak_ (mL.kg^-1^.min^_1^)	20.3 (7.5)	21.05 (7. 7)	0.9 (0.8; 1.0)	2.4	6.7	1.9
HR_peak_ (beats per minute)	125 (30)	126 (29)	0.9 (0.8; 1.0)	9.7	26.8	7.4
RER_peak_	0.9 (0.1)	0.9 (0.1)	0.7 (0.4; 0.9)	0.0	0.1	0.0
distance walked (meters)	593 (374)	632 (374)	0.97 (0.92; 0.99)	64.8	179.7	49.8
Number of shuttles completed	9.9 (5.3)	10.4 (5.5)	1.0 (0.9; 1.0)	0.8	2.1	0.6
1^st^VT (L.min^-1^)	1.7 (.4)	1.7 (.5)	0.8 (0.5; 1.0)	0.2	0.5	0.2
1^st^VT (mL.kg^-1^.min^-1^)	20.5 (5.0)	20.9 (3.9)	0.7 (0.1; 0.9)	2.6	7.1	2.0

HR_peak_ = peak Heart Rate, RER_peak_ - = peak Respiratory Exchange Rate, SEM = Standard error of Measurement, SDC = Smallest Detectable Change.

In Table 3 the results of the reproducibility of the Shuttle Test are presented with the single measure ICC_2,1 agreement_, SEM and SDC measures for the whole sample. VO_2peak_ L.min^-1^, VO_2peak_ mL.kg^-1^.min^-1^, number of shuttles and meters walked had ICCs > 0.90 and were considered excellent. For the reproducibility measures for the chronic people with stroke, see S1 Appendix and for the reproducibility measures for the subacute group, see S2 Appendix.

### Validity

For criterion validity we determined if the ST stresses the cardiopulmonary system sufficiently to attain at least the 1^st^ VT or VO_2max_ in at least 80% of participating participants. Ten (50%) out of the 20 participants attained 1^st^VT in the first test. The same ten participants and an additional two participants (60%) attained a 1^st^VT in the retest. One participant aged 79 years attained a RER = 1.04 in the test and one subject aged 65 years attained a RER = 1.0 in the retest, suggesting that by our criteria they reached VO_2max_.

Two participants attained the 1^st^VT at the end of both test and retest and three additional participants attained the 1^st^VT at the end of the retest. There were no significant differences between test and retest measures of 1^st^VT.

The mean difference (SE) in the lower extremity Motricity Index between participants who attained a 1^st^VT and those who did not was 10.4 (6.8) 95%CI -24.6; 3.8. In the retest the mean difference (SE) in the lower extremity Motricity Index was 14.5 (6.5), 95% CI -28.2; -0.9. In both instances the lower extremity Motricity Index was lower for those who did not attain a 1^st^VT, but this was only statistically significantly different in the retest.

**Fig 1:**
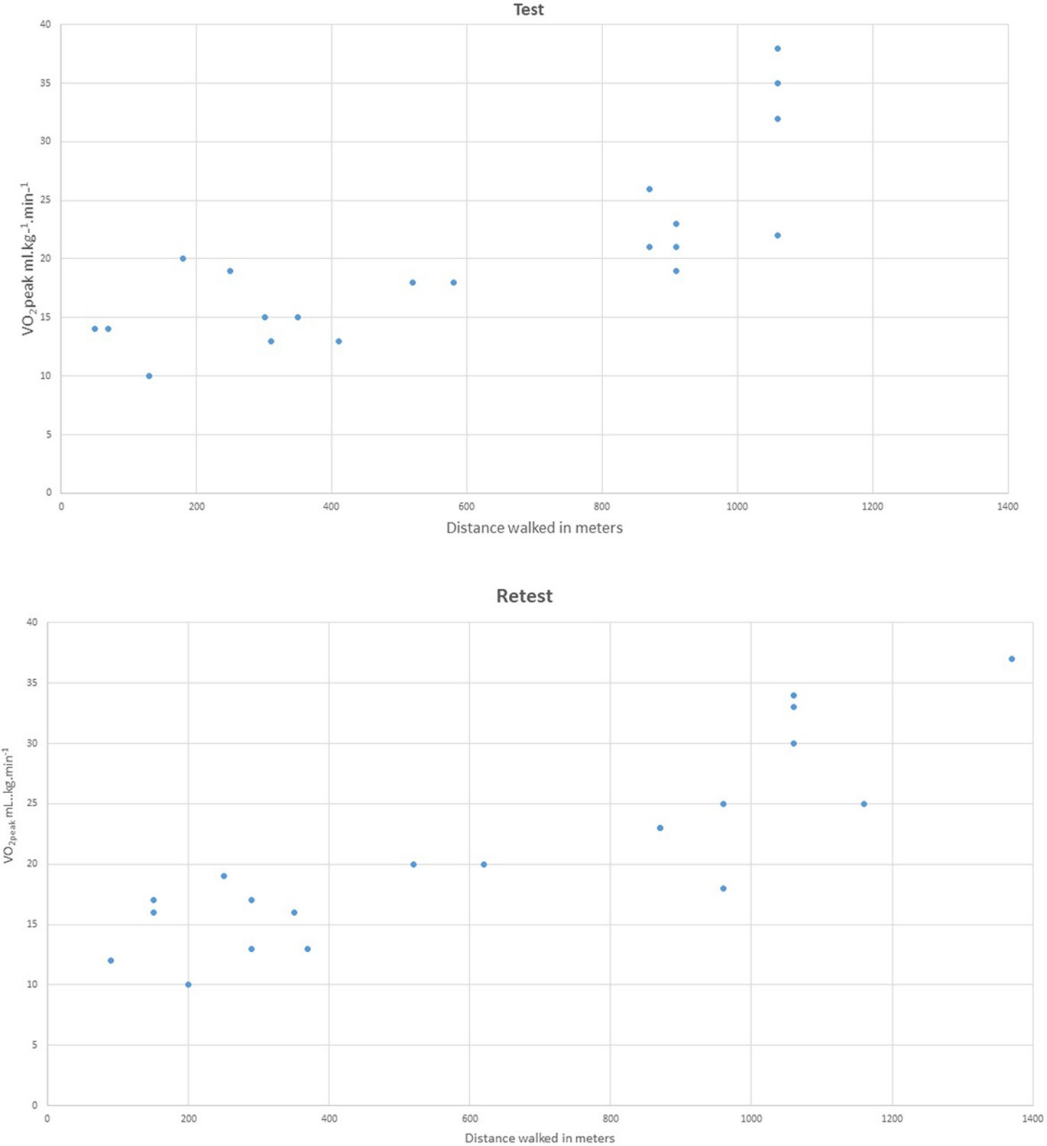
Scatterplot distance walked in meters and VO_2peak_ mL.kg.min^-1^ test and retest.

For convergent validity we found 2-sided Pearson correlations between number of shuttles completed and cardiovascular values were significant (p< 0.01) for VO_2peak_ L.min^-1^ r = 0.7 (test) and r = 0.8 (retest) and for VO_2peak_ mL.kg^-1^.min^-1^ r = 0.8 (test) and r = 0.9 (retest) (see Fig 1).

The total lower extremity Motricity Index had a significant association (p< 0.01) of r = 0.6 with the number of shuttles completed for the test and retest, respectively. There was no significant association of the number of shuttles with sex, body height or weight.

Forward multivariate analysis with the number shuttles completed at the first test as the dependent variable and lower extremity Motricity Index and VO_2peak_ ml.kg^-1^.min^-1^ as independent variables showed an adjusted R^2^ of 0.6 in the final model with VO_2peak_ mL.kg^-1^.min^-1^ (p<0.001).

At the retest, the adjusted R^2^ was 0.76 with lower extremity Motricity Index (p = 0.05) and VO_2peak_ mL.kg^-1^.min^-1^ (p = 0.001) contributing significantly to the model (see Table 4).

**Table 4:** Coefficients for number of shuttles (dependent variable) at retest.

Variables		Standardized Coefficients	t	Sig.	95% Confidence Interval for B	
		Beta			Lower Bound	Upper Bound
1	(Constant)		-1.237	0.23	-6.471	1.674
	RelVO_2peak_T2	0.855	7.006	0.001	0.426	0.790
2	(Constant)		-2.510	0.02	-15.674	-1.356
	RelVO_2peak_T2	0.743	6.011	0.001	0.343	0.714
	Lower extremity Motricity Index	0.262	2.115	0.05	0.001	0.182

### Differences in people with stroke in the subacute versus chronic phase

Seven in-patient rehabilitation (subacute) participants, 4 males and 3 females, were included in the sample. Independent t-testing between the subacute sample and the sample with chronic stroke revealed the subacute sample was significantly younger (mean difference (SE) = 20 (6) years, 95% 7.1;33.0) Their median lower extremity Motricity Index was significantly higher (p = 0.007). The mean difference (SE) in number of meters walked (327.2 (162.9), 95% CI -669.5; 15.2) did not significantly differ between groups in the test, but was significantly higher in the retest 378.6 (175.90 meters, 95% CI -748.2; -8.9. VO_2peak_ L.min^-1^ did not differ between the groups. In contrast, the mean difference (SE) VO_2peak_ ml.kg^-1^.min^-1^ was significantly higher in both test 9.0 (3.6) ml.kg^-1^.min^-1^, 95% CI -17.4;-0.5 and retest 9.2 (3.7) ml.kg^-1^.min^-1^, 95% CI -17.6; -0.7 for the subacute sample. For details, see S3 Appendix.

## Discussion

This study provides insight into the cardiopulmonary response to the 10mST in people with stroke both earlier (within 3 months) and later (≥ 6 months post-stroke). None of the participants were able to complete the supramaximal test, suggesting they reached maximal exertion in the 10mST. Therefore, the 10mST can be considered a symptom limited maximal exercise test for people with stroke. The metabolic demand of the 10mST varied widely as the primary limiting factor was motor performance for most participants.

The 10mST is a safe, feasible and reproducible test for measuring symptom limited maximal exercise capacity with good convergent validity people with stroke. Criterion validity, however, indicates it is not a valid test for measuring maximal cardiopulmonary capacity. Eighty-five percent of participants in the test-retest sample stopped due to motor and/or coordination problems. By our criteria, only two participants (5%) achieved a VO_2max_ in the test or retest; both of these participants had minimal motor impairments. A 1^st^VT could only be determined in 50% of participants during the first test and in 60% during the second test.

For convergent validity we found a good to excellent relationship between the number of shuttles completed and cardiopulmonary capacity. This was confirmed in the multiple regression analysis where VO_2peak_ mL.kg^-1^.min^-1^ made a significant contribution to the number of shuttles completed. Participants with higher cardiopulmonary capacity were able to walk more shuttles.

Reproducibility was consistent with the results reported by van Bloemendaal et al. [12] who reported similar ICC_2,1 agreement_ scores, but a higher standard error of measurement (109 m, compared with 65 m in our study. Similar to Bongers et al. [31] in our study all retest measures were slightly higher, which suggests a learning effect. This is in contrast to Bloemendaal et al. [12] who did not find a learning effect. Nevertheless, a habituation session prior to the 10mST is recommended.

The finding that those with lower lower extremity Motricity Index (i.e. less strength) were less likely to reach a 1^st^VT, matches the participants’ reporting stopping due to motor / coordination problems. This is in contrast to Marzolini et al. [32] who reported 76% of participants had a discernible 1^st^VT during a maximal exercise test on a recumbent bicycle, and did not differ significantly from those who did not reach a 1^st^VT by lower limb motor impairment and balance score.

### Study strengths and weaknesses

This is the first study to demonstrate reproducibility of a field-based cardiopulmonary capacity / fitness test in people with stroke [4]. However, our sample size was small and six participants (23%) of the initial sample were unable to perform the first level of the 10mST. Apart from being very slow walkers (< 2 km/h or 0.56 m/s), they also had significantly less lower extremity motor strength and balance. The 10mST may therefore not be a useful test for very slow walkers (those walking 2 km/h or slower) or for people with severe balance problems (those scoring 11 or less on the POMA).

Prior research showed balance, measured by the POMA, has a confounding effect on distance walked in the 6MWT [33], but we did not find this effect in this study. As we excluded participants with a history of falls, potentially excluding those with poorer balance, we may have limited this confounding effect. This could also have positively affected the number of shuttles completed. We did not measure the distance walked, but estimated it from the number of shuttles walked. This could have resulted in overly optimistic reproducibility measures for the distance walked.

### Implications

Walk tests, such as the commonly used 6-minute walk test (6MWT), have been designed and used as surrogate measures of cardiorespiratory capacity in other populations, but associations between distance walked and cardiopulmonary capacity are low to moderate in people with stroke [10]. Correlations between the 10mST and cardiopulmonary capacity were higher (r>0.7) than those reported for the 6MWT (mean r = 0.5, 95% CI 0.4–0.6) [10], suggesting the 10mST may be a better measure for assessing cardiopulmonary capacity than the 6MWT.

In addition, the 10mST is a maximal symptom limited exercise test and may be useful as a screener for exercise tolerance and the potential adverse effects of exercise.

Further studies should examine the responsiveness and minimally important change in this population to determine if the test can be used as an outcome measure.

### Clinical messages

- The 10mST is a maximal symptom limited exercise test
- The 10mST is a safe, feasible and reproducible test in people with subacute and chronic stroke
- The 10mST has a good to excellent relationship with cardiopulmonary capacity

## Supporting information

S1 Appendix Reproducibility 10mST chronic people with stroke. (DOCX)

S2 Appendix Reproducibility 10mST subacute people with stroke. HR_peak_ = peak Heart Rate, RER_peak_ - = peak Respiratory Exchange Rate, SEM = Standard error of Measurement, SDC = Smallest Detectable Change.
(DOCX)

S3 Appendix Differences in test results between persons with stroke in the subacute and chronic phase. (DOCX)

## References

[1] World Health Organisation. Ageing and life-course. What is healthy ageing? [Internet]. online. [cited 2020 Jun 30]. Available from: https://www.who.int/ageing/healthy-ageing/en/

[2] Eng JJ, Dawson AS, Chu KS. Submaximal exercise in persons with stroke: test-retest reliability and concurrent validity with maximal oxygen consumption. Arch Phys Med Rehabil. 2004 1;85(1):113–8. 10.1016/s0003-9993(03)00436-2 14970978 PMC3167868

[3] Mackay-Lyons MJ, Makrides L. Exercise capacity early after stroke. Arch Phys Med Rehabil. 2002 12;83(12):1697–702. 10.1053/apmr.2002.36395 12474172

[4] Wittink H, Verschuren O, Terwee C, de Groot J, Kwakkel G, van de Port I. Measurement properties of maximal cardiopulmonary exercise tests protocols in persons after stroke: A systematic review. J Rehabil Med. 2017 11;49(9):689–99. 10.2340/16501977-2260 28862313

[5] Boyne P, Reisman D, Brian M, Barney B, Franke A, Carl D, et al Ventilatory threshold may be a more specific measure of aerobic capacity than peak oxygen consumption rate in persons with stroke. Top Stroke Rehabil. 2017 3;24(2):149–57. 10.1080/10749357.2016.1209831 27454553 PMC5588902

[6] Tang A, Sibley KM, Bayley MT, McIlroy WE, Brooks D. Do functional walk tests reflect cardiorespiratory fitness in sub-acute stroke? J Neuroeng Rehabil. 2006;3:23 10.1186/1743-0003-3-23 17010203 PMC1592502

[7] Eng JJ, Chu KS, Dawson AS, Kim CM, Hepburn KE. Functional walk tests in individuals with stroke: relation to perceived exertion and myocardial exertion. Stroke. 2002 3;33(3):756–61. 10.1161/hs0302.104195 11872900

[8] Salbach NM, Brooks D, Romano J, Woon L, Dolmage TE. Cardiorespiratory responses during the 6-minute walk and ramp cycle ergometer tests and their relationship to physical activity in stroke. Neurorehabil Neural Repair. 2014 2;28(2):111–9. 10.1177/1545968313498826 23921421

[9] Pang MYC, Eng JJ, Dawson AS. Relationship between ambulatory capacity and cardiorespiratory fitness in chronic stroke: influence of stroke-specific impairments. Chest. 2005 2;127(2):495–501. 10.1378/chest.127.2.495 15705987 PMC4820333

[10] Outermans J, van de Port I, Wittink H, de Groot J, Kwakkel G. How strongly is aerobic capacity correlated with walking speed and distance after stroke? Systematic review and meta-analysis. Phys Ther. 2015;95(6). 10.2522/ptj.20140081 25573761

[11] Verschuren O, Takken T, Ketelaar M, Gorter JW, Helders PJM. Reliability and validity of data for 2 newly developed shuttle run tests in children with cerebral palsy. Phys Ther. 2006 8;86(8):1107–17. 16879044

[12] van Bloemendaal M, Kokkeler AM, van de Port IG. The shuttle walk test: a new approach to functional walking capacity measurements for patients after stroke? Arch Phys Med Rehabil. 2012 1;93(1):163–6. 10.1016/j.apmr.2011.08.012 22200397

[13] Stroke—1989. Recommendations on stroke prevention, diagnosis, and therapy. Report of the WHO Task Force on Stroke and other Cerebrovascular Disorders. Stroke. 1989 Oct;20(10):1407–31. 10.1161/01.str.20.10.1407 2799873

[14] Bernhardt J, Hayward KS, Dancause N, Lannin NA, Ward NS, Nudo RJ, et al A Stroke Recovery Trial Development Framework: Consensus-Based Core Recommendations from the Second Stroke Recovery and Rehabilitation Roundtable. Neurorehabil Neural Repair. 2019 11;33(11):959–69. 10.1177/1545968319888642 31674274

[15] Riebe D, Franklin BA, Thompson PD, Garber CE, Whitfield GP, Magal M, et al Updating ACSM’s Recommendations for Exercise Preparticipation Health Screening. Med Sci Sports Exerc. 2015 11;47(11):2473–9. 10.1249/MSS.0000000000000664 26473759

[16] Aly N and Mailk IH. Appoaching geriatric patients: the frequent fallers. Scottish Univ Med J. 2013;2:20–7.

[17] Lamb SE, Jorstad-Stein EC, Hauer K, Becker C. Development of a common outcome data set for fall injury prevention trials: the Prevention of Falls Network Europe consensus. J Am Geriatr Soc. 2005 9;53(9):1618–22. 10.1111/j.1532-5415.2005.53455.x 16137297

[18] World Medical Association. World Medical Association Declaration of Helsinki: ethical principles for medical research involving human subjects. JAMA. 2013 11;310(20):2191–4. 10.1001/jama.2013.281053 24141714

[19] American College of Sportsmedicine. ACSM’s guidelines for exercise testing and prescription. 6th ed. Philadelphia: Lippincott Williams and Wilkins; 2000.

[20] Heidari S, Babor TF, Castro P De, & ST, Curno M. Sex and Gender Equity in Research: rationale for the SAGER guidelines and recommended use. BMC Res Integr Peer Rev [Internet]. 2016;1(2). Available from: https://kopernio.com/viewer?doi=10.1186/s41073-016-0007-6&route=6 29451543 10.1186/s41073-016-0007-6 PMC5793986

[21] Tinetti ME. Performance-oriented assessment of mobility problems in elderly patients. J Am Geriatr Soc. 1986 2;34(2):119–26. 10.1111/j.1532-5415.1986.tb05480.x 3944402

[22] Demeurisse G, Demol O, Robaye E. [Motor evaluation in vascular hemiplegia]. Brux Med. 1979 2;59(2):87–94. 427624

[23] Vogler AJ, Rice AJ, Gore CJ. Validity and reliability of the Cortex MetaMax3B portable metabolic system. J Sports Sci. 2010 5;28(7):733–42. 10.1080/02640410903582776 20419553

[24] Overstreet BS, Bassett DRJ, Crouter SE, Rider BC, Parr BB. Portable open-circuit spirometry systems. J Sports Med Phys Fitness. 2017 3;57(3):227–37. 10.23736/S0022-4707.16.06049-7 26861831

[25] Nolan PB, Beaven ML, Dalleck L. Comparison of intensities and rest periods for VO2max verification testing procedures. Int J Sports Med. 2014 11;35(12):1024–9. 10.1055/s-0034-1367065 24886925

[26] Rossiter HB, Kowalchuk JM, Whipp BJ. A test to establish maximum O2 uptake despite no plateau in the O2 uptake response to ramp incremental exercise. J Appl Physiol. 2006 3;100(3):764–70. 10.1152/japplphysiol.00932.2005 16282428

[27] Edvardsen E, Scient C, Hansen BH, Holme IM, Dyrstad SM, Anderssen SA. Reference values for cardiorespiratory response and fitness on the treadmill in a 20- to 85-year-old population. Chest. 2013 7;144(1):241–8. 10.1378/chest.12-1458 23287878

[28] Binder RK, Wonisch M, Corra U, Cohen-Solal A, Vanhees L, Saner H, et al Methodological approach to the first and second lactate threshold in incremental cardiopulmonary exercise testing. Eur J Cardiovasc Prev Rehabil. 2008 12;15(6):726–34. 10.1097/HJR.0b013e328304fed4 19050438

[29] Terwee CB, Bot SDM, de Boer MR, van der Windt DAWM, Knol DL, Dekker J, et al Quality criteria were proposed for measurement properties of health status questionnaires. J Clin Epidemiol. 2007 1;60(1):34–42. 10.1016/j.jclinepi.2006.03.012 17161752

[30] Portney L.G., Watkins MP. Foundations of clinical research: applications to practice. 2nd ed. Prentice Hall; New Jersey; 2000 1–848 p.

[31] Bongers BC, Rijks EBG, Harsevoort AGJ, Takken T, van Brussel M. 10-m Shuttle Ride Test in Youth With Osteogenesis Imperfecta Who Use Wheelchairs: Feasibility, Reproducibility, and Physiological Responses. Phys Ther. 2016 5;96(5):679–86. 10.2522/ptj.20150082 26494769

[32] Marzolini S, Oh P, Corbett D, Dooks D, Calouro M, MacIntosh BJ, et al Prescribing Aerobic Exercise Intensity without a Cardiopulmonary Exercise Test Post Stroke: Utility of the Six-Minute Walk Test. J Stroke Cerebrovasc Dis. 2016 9;25(9):2222–31. 10.1016/j.jstrokecerebrovasdis.2016.04.016 27289183

[33] Outermans JC, van de Port I, Kwakkel G, Visser-Meily JM, Wittink H. The role of postural control in the association between aerobic capacity and walking capacity in chronic stroke: a cross-sectional analysis. Eur J Phys Rehabil Med. 2018 3; 10.23736/S1973-9087.18.04987-0 29532648

