# Supplementary material for: Feasibility, reproducibility and validity of the 10 meter Shuttle Test in mild to moderately impaired people with stroke
Source: PLoS One. 2020 Oct 28;15(10):e0239203. doi: 10.1371/journal.pone.0239203 (PMC7592795; doi:10.1371/journal.pone.0239203)
Supplement: S1 Appendix — (DOCX) [file pone.0239203.s001.docx]

S1 Appendix. Reproducibility 10mST chronic people with stroke

| Mean (SD) | Test  (n=13) | Retest  (n=13) | Single measure ICC_1,2_ agreement  95% CI | SEM | SDC_ind_ | SDC_group_ |
| --- | --- | --- | --- | --- | --- | --- |
| VO_2peak_ (L.min^_1^) | 1.4 (0.6) | 1.5 (0.6) | 0.9 (0.8; 1.0) | 0.2 | 0.4 | 0.1 |
| VO_2peak_  (mL.kg^-1^.min^_1^) | 17.2(4.2) | 17.9 (4.5) | 0.9 (0.6 ;1.0) | 1.6 | 4.5 | 13 |
| HR_peak_ beats per minute | 116 (26) | 119 (24) | 0.9 (0.7; 1.0) | 7.5 | 20.7 | 5.8 |
| RER_peak_ | 0.9 (0.1) | 0.9 (0.1) | 0.6 (0.1; 0.9) | 0.1 | 0.1 | 0.0 |
| Distance walked (meters) | 478.5 (325.0) | 500.0 (321.3) | 1.0 (1.0; 1.0*) | 32.3 | 89.6 | 24.8 |
| Number of shuttles completed | 8.4 (4.9) | 8.8 (4.6) | 1.0 (0.9; 1.00) | 0.7 | 1.9 | 0.5 |
| 1^st^VT (L.min^-1^) | 1.7 (0.4) | 1.8 (0.5) | 0.7 (0.8; 1.0) | 0.2 | 0.6 | 0.2 |
| 1^st^VT  (mL.kg^-1^.min^-1^) | 18.2 (2.6) | 19.8 (3.4) | 0.4 (-0.4; 0.9) | 2.4 | 6.6 | 1.8 |

HR_peak_ = peak Heart Rate, RER_peak_ -= peak Respiratory Exchange Rate, SEM = Standard error of Measurement, SDC= Smallest Detectable Change *rounded up
