# Supplementary material for: Feasibility, reproducibility and validity of the 10 meter Shuttle Test in mild to moderately impaired people with stroke
Source: PLoS One. 2020 Oct 28;15(10):e0239203. doi: 10.1371/journal.pone.0239203 (PMC7592795; doi:10.1371/journal.pone.0239203)
Supplement: S2 Appendix — HRpeak = peak Heart Rate, RERpeak - = peak Respiratory Exchange Rate, SEM = Standard error of Measurement, SDC = Smallest Detectable Change. (DOCX) [file pone.0239203.s002.docx]

S2 Appendix. Reproducibility 10mST subacute people with stroke

| Mean (SD) | Test  ( n=7) | Retest  (n=7) | Single measure ICC_1,2_ agreement  (95% CI) | SEM | SDC_ind_ | SDC_group_ |
| --- | --- | --- | --- | --- | --- | --- |
| VO_2peak_ (L.min^_1^) | 1.8 (0.8) | 1.9 (0.9) | 0.9 (0.7; 1.0) | 0.2 | 0.6 | 0.2 |
| VO_2peak_  (mL.kg^-1^.min^_1^) | 26.1 (9.1) | 27.0 (9.1) | 0.9 (0.4; 1.0) | 3.4 | 9.4 | 3.6 |
| HR_peak_ beats per minute | 141 (32) | 137 (35) | 0.8 (0.4; 1.0) | 13.2 | 36.7 | 13.9 |
| RER_peak_ | 0.9 (0.1) | 1.0 (0.1) | 0.8 (0.2; 1.0) | 0.0 | 0.1 | 0.0 |
| Distance walked (meters) | 805 (388) | 878 (464) | 0.9 (0.7; 1.0) | 105 | 291 | 110 |
| Number of shuttles completed | 12.7 (6.0) | 13.4 (6.0) | 1.0 (0.8; 1.0) | 1.2 | 3.3 | 1.3 |
| 1^st^VT (L.min^-1^) | 1.6 (0.5) | 1.4 (0.5) | 1.0 (0.6; 1.0) | 0.1 | 0.3 | 0.1 |
| 1^st^VT  (mL.kg^-1^.min^-1^) | 24.0 (5.9) | 21.0 (4.2) | 0.8 (-.3; 1.0) | 2.6 | 7.2 | 2.7 |

HR_peak_ = peak Heart Rate, RER_peak_ -= peak Respiratory Exchange Rate, SEM = Standard error of Measurement, SDC= Smallest Detectable Change
