# Supplementary material for: Feasibility, reproducibility and validity of the 10 meter Shuttle Test in mild to moderately impaired people with stroke
Source: PLoS One. 2020 Oct 28;15(10):e0239203. doi: 10.1371/journal.pone.0239203 (PMC7592795; doi:10.1371/journal.pone.0239203)
Supplement: S3 Appendix — (DOCX) [file pone.0239203.s003.docx]

S3 Appendix: Differences in test results between persons with stroke in the subacute and chronic phase

|  | Subacute  (n=7) | Chronic  (n=13) | p-value |
| --- | --- | --- | --- |
| TEST VO_2peak_ (L.min^_1^, mean (SD) | 1.8 (0.8) | 1.4 (0.6) | 0.23* |
| RETEST VO_2peak_ (L.min^_1^, mean (SD)) | 1.9 (0.9) | 1.5 (0.6) | 0.24* |
| TEST VO_2peak_ (ml.kg^-1^.min^_^1, mean (SD)) | 26.1 (9.1) | 17.2 (4.2) | **0.04*** |
| RETEST VO_2peak_ (ml.kg^-1^.min^_1^, mean (SD)) | 27.0 (9.1) | 17.9 (4.5) | **0.04*** |
| VO_2peak_ (ml.kg^-1^.min^_1^, mean (SD))  TEST  RETEST | 26.1 (9.1)  27.0 (9.1) | 17.2 (4.2)  17.9 (4.5) | **0.04***  **0.04*** |
| HR_peak bpm_ (mean (SD))  TEST  RETEST | 140.7 (32.0)  137.4 (34.5) | 116.1 (25.9)  119.1 (23.9) | 0.08*  0.17* |
| RER_peak_ (mean (SD))  TEST  RETEST | 0.9 (0.1)  1.0 (0.1) | 0.9 (0.0)  0.9 (0.1) | 0.25*  0.49* |
| distance walked (meters, (mean (SD)) TEST  RETEST | 805.7 (388.8)  878.6 (464.9) | 478.5 (325.0) 500.0 (321.3) | 0.06*  **0.05*** |
| number of shuttles (mean (SD))  TEST  RETEST | 12.7 (5.2)  13.4 (6.0) | 8.4 (4.9)  8.8 (4.6) | 0.08*  0.07* |
| Borg after test (median (range))  TEST  RETEST | 15 (7-17)  17 (7-18) | 15 (7-17)  16 (8-18) | 0.82**  0.88** |
| Reason for stopping (n (%))  TEST  Out of breath /tired  Motor limitation/ Coordination  RETEST  Out of breath /tired  Motor limitation/ Coordination | 1 (14.3)  6 (85.7)  0  7 (100) | 4 (30.8)  9 (69.2)  5 (41.7)  7 (58.3) | 0.56***  0.23*** |
| VT reached (Yes, n (%))  TEST  RETEST | 4 (57.1)  6 (85.7) | 6 (46.2)  6 (46.2) | 0.64***  0.09*** |
| 1^st^VT (ml.kg^-1^.min^_1^, mean(SD))  TEST  RETEST | 24.0 (5.9)  22.5 (4.5) | 18.2 (2.6)  19.8 (3.4) | 0.06*  0.61* |

*Independent t-test, **Mann-Whitney U test, ***Chi square
