# Supplementary material for: Feasibility, reproducibility and validity of the 10 meter Shuttle Test in mild to moderately impaired people with stroke
Source: PLoS One. 2020 Oct 28;15(10):e0239203. doi: 10.1371/journal.pone.0239203 (PMC7592795; doi:10.1371/journal.pone.0239203)
Supplement: Attachment — Submitted filename: Response to reviewers.docx [file pone.0239203.s004.docx]

April 22nd, 2020

Dear dr. Gerson Cipriano Jr., PT, MsC, Ph.D ( and reviewers)

We would like to express our sincere gratitude to the reviewers for spending their time and effort in reviewing our manuscript. We feel that the comments and recommendations made by the expert reviewers has helped us to improve the quality of our paper. The manuscript has been revised following each of the recommendations. Please see our detailed responses to the comments (marked as [reply]) below. Please also refer to the highlighted sentences in yellow for changes that have been made in the manuscript.

Reviewer #1:

1. While the authors are aware of the study aspects that have potentially limited the generalization of findings, they fail to adequately present the study population in both the title of the manuscript and the conclusion. It should be made clear to readers that the reported findings are only valid in “highly functioning stroke survivors” or another similar, more specific possibility.

[reply] That is a good point. Thank you. In the title we now included “Mild to moderately impaired Stroke survivors”.

2. Two independent raters determined measures of 1st VT. Yet, no interrater reliability data have been reported. Please analyze and provide the degree of agreement among the examiners.

[reply] In lines 185-187 we added: We calculated two way random Intraclass Correlation Coefficients with absolute agreement for the interrater reliability of 1^st^ VT and found it to be ICC_2,1_=0.91 with a 95% CI .38 -.98.

3. The authors use the term “reproducibility” throughout the text when, in fact, this should be named “repeatability” or even "test-retest reliability". There is a difference between reproducibility and repeatability.

[reply] Thank you for your question. However, we do not agree with your suggestion/remark. Reproducibility comprises both reliability and agreement.(de Vet et al. 2006) Reliability was measured by intraclass correlation coefficients (ICC, two-way mixed). In the case of agreement, the standard errorof measurement (SEM) was used to determine the precision of the total score of both tests. The SEM describes the error in interpreting the test score of an individual. Moreover, this terminology is also used by what is now called the COSMIN risk of bias tool. Although this was initially developed to assess the quality of research on questionnaires, it has also been used to assess the quality of measurement studies into physical tests, such as the 6 minute walk test, with permission and consent of the COSMIN authors.
Reproducibility concerns the degree to which repeated measurements in stable persons (test-retest) provide similar answers. Terwee et al. believe that it is important to make a distinction between reliability and agreement and this distinction is used throughout the COSMIN risk of bias tool which is now the Gold standard for assessing measurement properties. Agreement concerns the absolute measurement error, i.e., how close the scores on repeated measures are, expressed in the unit of the measurement scale at issue. Small measurement error is required for evaluative purposes in which one wants to distinguish clinically important changes from measurement error. Reliability concerns the degree to which patients can be distinguished from each other, despite measurement error [19]. High reliability is important for discriminative purposes if one wants to distinguish among patients, e.g., with more or less severe disease (as in diagnostic applications).

4. There are two types of construct validity but the authors only report on one of them: convergent validity. Please use this term instead.
[reply] According to the COSMIN: https://www.cosmin.nl/wp-content/uploads/COSMIN_taxonomy.pdf, there are three types of construct validity:

- Structural validity (factor structure)
- Cross-cultural validity
- Hypothesis testing (convergent validity)

Both structural validity and cross-cultural validity we deemed Not Applicable in this study. We replaced “construct” validity with “convergent” throughout the document.

5. Still on the convergent validity analysis: please provide scatter plots to illustrate the most significant relationships observed.

[reply] We added a scatterplot of VO_2peak_ mL.kg.min^-1^ and distance walked in meters (Figure 1.) for the test and retest, see line 297 and page 37.

[for the editor] we uploaded this image in Pace.

6. Bland-Altman plots should have been provided and used to determine if any systematic differences across the range of values occurred between the two testing sessions.

[reply] We did not provide Bland-Altman plots as we had a small sample size and the 95% CI would be very large, giving much uncertainty about accuracy of the limits of agreement. Bland and Altman recommend providing plots in sample sizes >100 only and preferably in N>200. Please see: <https://www-users.york.ac.uk/~mb55/meas/sizemeth.htm>

Minor edits and questions:

7. Please define “close to completion” (line 112, p. 6)

[reply] We refer to those patients who were about ready for discharge from the rehabilitation centre. On line 110-112 we added: …and be close to completion of their in-patient rehabilitation (at the rehabilitation centre), and about to be discharged from the rehabilitation centre.

8. Please specify how the “recurrent faller” info was obtained (i.e. medical records, self-reported…)

[reply] We have added to the text: Sex and recurrent falls were defined based on self-report ^21^ (line 134)

9. Was a gait belt or any other device used for support during the tests?

[reply] In line 148-149 we added: No gait belts or other devices were used for support, but patients were allowed to use their own assistive devices.

10. Why was a 20-minute recovery period chosen? I wonder whether this was enough time for them to fully recover.

[reply] We based our choice on the following reference that is used in this section in the manuscript: Nolan PB, Beaven ML, Dalleck L. Comparison of intensities and rest periods for VO2max verification testing procedures. *Int J Sports Med* 2014;35:1024–9 and our own experience in maximal exercise testing in stroke survivors. The 20 minutes turned out to be enough as most patients were limited by their motor function and did not exercise to their cardiovascular limits.

11. Were individuals asked to withdraw medications for the tests?

[reply] No, we did not ask patients to withdraw medications for the test. This, because we were dealing with a vulnerable population and heart rate nor blood pressure were primary outcome parameters.

12. Please clarify “time of testing”. What do you mean? Daytime vs. nighttime?

[reply] Time of testing refers to test 1 versus test 2 or test versus retest – see line 205

13. Reliability info was provided for the entire sample and for the subacute group, but not for the chronic group specifically.

[reply] We added a Table with reproducibility measures for the chronic sample in the Appendices, see S1 Appendix. And added in lines 273-274: For the reproducibility measures for the chronic stroke survivors, see S1 Appendix and for the reproducibility measures for the subacute group, see S2 Appendix.

Reviewer #2:

Areas of Primary Concern:
-This study used as exclusion criteria patients with more significant balance dysfunction as defined by a history of “recurrent falls” (was this assessed via self report?), and then a total of 6 out of 26 recruited subjects did not complete the entire study due to the inability to complete the first stage of the test, thus reducing the overall sample population assessed to those with subacute/chronic stroke and higher levels of walking ability. This was discussed within the body paper, yet the title of the paper could be misleading in that it seems to indicate that the study population was all “stroke survivors”. Please consider re-writing the title to indicate the specific nature of the study population.

[reply] In the title we now included “Mild to moderately impaired Stroke survivors”.


-The authors use the term reproducibility throughout the paper regarding the stability of results with administration of the outcome measure in the same patients on two different occasions separate by a time period, but perhaps what they mean is test-retest reliability or repeatability?

[reply] Reproducibility concerns the degree to which repeated measurements in stable persons (test-retest) provide similar answers. Terwee et al. believe that it is important to make a distinction between reliability and agreement and this distinction is used throughout the COSMIN risk of bias tool which is now the Gold standard for assessing measurement properities.

Agreement concerns the absolute measurement error, i.e., how close the scores on repeated measures are, expressed in the unit of the measurement scale at issue. Small measurement error is required for evaluative purposes in which one wants to distinguish clinically important changes from measurement error. Reliability concerns the degree to which patients can be distinguished from each other, despite measurement error. High reliability is important for discriminative purposes if one wants to distinguish among patients, e.g., with more or less severe disease (as in diagnostic applications).

-It is not reported whether there was any assessment of tone in this study, which is a variable that may be likely to have an effect or interaction with the outcomes and would have been interesting to include in the baseline assessments of the patient population.

[reply] In our understanding tone/spasticity is a clinical symptom of the loss of motor control. We assessed motor control as part of the construct of functional strength using the Motricity Index. Another argument to not solely asses tone is the low reproducibility of clinical measures such as de Modified Ashworth Scale (MAS) or Tardieu scale, which would probably produce unreliable results.

-It was reported in the data tables how many patients used assistive devices out of the overall sample, but it was not reported whether these patients used their assistive devices during the performance of the shuttle test.

[reply] Thank you, we amended this in the text: In line 148-149 we added: No gait belts or other devices were used for support, but patients were allowed to use their own assistive devices.

-Line 76: Please consider re-wording this statement: The wording regarding “cardiac vs non-cardiac” exercise limitation should probably be rephrased to state that inability to reach the 1st VT only helps you distinguish whether the patient is limited due to their capacity to sustain adequate aerobic metabolism to support a given exercise test, versus being limited by another reason (in the case of this patient population, motor control/neuromuscular function). It is not sensitive enough to distinguish between the dysfunction in aerobic capacity that could be attributed to a particular organ system (i.e. cardiac vs pulmonary) as this sentence seems to indicate.

[reply] Thank you, we removed the sentence.

-Please review the total summation of the numbers of people using assistive device in Table 1 starting at line 609 “Assistive devices indoors”, the totals seem to be off by 1

[reply] Thank you for noticing! Oeps! We corrected the Table.

-Would like to have seen a 6MWT performed so that those results could be related to this study population, and since the 6MWT is the only other highly commonly used outcome measure in clinical practice. Especially since this study population was so filtered to include higher-functioning individuals with better balance. Perhaps the ST only looks like a more reliable measure because the study population was filtered in this way? We are unable to compare these results to other studies that have looked at 6MWT.

[reply] We agree with you. In fact, the subacute sample was functioning at quite a high level, but the chronic sample did not function all that well, considering the amount of supportive devices they use when outdoors. The problem is that although the 6 min walk test is well established it it not a measure of cardiopulmonary capacity and the correlations of distance walked with _VO2peak_ are not all that great. It would be good to repeat this study with a (larger) sample of community ambulating stroke survivors and include those with less than good balance. We are hoping this might be our next step ☺.

On behalf of all authors,

Yours sincerely,

Dr. Harriet Wittink
