# Supplementary material for: Feasibility, reproducibility and validity of the 10 meter Shuttle Test in mild to moderately impaired people with stroke
Source: PLoS One. 2020 Oct 28;15(10):e0239203. doi: 10.1371/journal.pone.0239203 (PMC7592795; doi:10.1371/journal.pone.0239203)
Supplement: Attachment — Submitted filename: Editor_SWT_revised manuscript with track changes.docx [file pone.0239203.s005.docx]

**Feasibility, reproducibility and validity of the 10 meter Shuttle Test in mild to moderately impaired stroke survivors**.

Wittink, H^1*^, Blatter, T^1^, Outermans, J^1^, Volkers, M^2^, Westers, P^3^, Verschuren, O^2.^

1. Research group Lifestyle and Health, Utrecht University of Applied Sciences, Utrecht, the Netherlands

2. UMC Utrecht Brain Center and Center of Excellence for Rehabilitation Medicine, Utrecht University, Utrecht, The Netherlands,

3. Julius Centre for Health Sciences and Primary Care, University Medical Center Utrecht, Utrecht, The Netherlands

*Corresponding author

**Abstract**

**Background:** There currently is no field test available for measuring maximal exercise capacity in stroke survivors

**Objective:** to determine the feasibility, reproducibility and validity of the Shuttle Test (ST) to measure exercise capacity in stroke survivors.

**Design:** Longitudinal study design

**Setting:** rehabilitation department, day care centres from a nursing home and private practices specialized in neuro rehabilitation.

**Subjects:** People with subacute or chronic stroke

**Interventions**: A standardized protocol with a portable gas analyser was used to determine feasibility, reproducibility and validity of the 10-meter Shuttle Test

**Main Measures:** Number of shuttles walked, 1^st^VT

**Results:** The Shuttle Test is a safe, feasible and reproducible test for measuring maximal exercise capacity with good convergent validity for cardiopulmonary capacity in subacute and chronic stroke survivors. Criterion validity, however, indicates it is not a valid test for measuring maximal cardiopulmonary capacity (VO_2max_). Only 60% of participants were able to reach the 1^st^VT. The associations of the number of shuttles walked and cardiopulmonary capacity were r > 0.72. Higher cardiopulmonary capacity and a higher total score of the lower extremity Motricity Index contributed significantly to a higher number of shuttles walked.

**Conclusions:** . As a field test, usable in most out-patient physiotherapy clinics, the Shuttle Test appears a useful maximal symptom limited exercise test except for very slow walkers.

**Key words:** Shuttle Test, cardiopulmonary capacity, stroke, Motricity Index, maximal exercise test.

**Word count:**

**Introduction**

Rehabilitation of stroke survivors is aimed at gaining as much independence as possible in their own environment. After discharge from the hospital stroke survivors need to stay or become physically active to keep or regain their cardiopulmonary capacity as an important part of integration into society^1^ and to remain healthy^2^.

The assessment of maximal cardiopulmonary capacity in stroke survivors is more challenging than in healthy subjects because they present with stroke-specific impairments such as muscle weakness, fatigue, poor balance, contractures and spasticity, which can compromise cardiopulmonary exercise test (CPET) outcome^3,4^. Given the reported difficulty of measuring VO_2max_ in stroke survivors^5^, we may need to consider alternatives, for instance, the determination of the first ventilatory threshold (1^st^VT). The 1^st^VT represents the intensity limit of prolonged exercise above which a transition to anaerobic metabolism begins^6^. As the intensity of exercise begins to increase, the 1^st^VT can be identified at the point where the breathing rate begins to increase. It is recommended as an appropriate target intensity level for the prescription of light to moderate exercise. It has been suggested the 1^st^VT may be a more specific measure of cardiopulmonary capacity than peak oxygen uptake in stroke survivors^6^. The 1^st^VT is a useful measure as work below this level encompasses most daily living activities.

Apart from the challenges of CPET in stroke survivors, it requires expensive equipment and trained personnel, not available to many clinicians. Consequently, there is an urgent need for a valid and reproducible field test to assess at least the 1^st^VT and if possible, VO_2max_. In clinical settings field tests of walking ability such as the six-minute walk test (6MWT) are often used. Prior studies have concluded that the 6MWT is not an adequate measure of cardiopulmonary fitness after stroke ^7–10^.

The 10 meter Shuttle Test (10mST), as modified for people with Cerebral Palsy (CP)^11^, may be of more use. For many adults with physical disabilities, the original 20-m shuttle test is not suitable, because the starting speed (8 km/h) and increase (0.5 km/h) every minute are beyond their capabilities. The adapted protocol, with a slower starting speed and smaller speed increases, has been found to be safe and to have acceptable reproducibility and convergent validity for children with CP^11^.

The ST has been found to have high reproducibility and validity when compared with the 6MWT in people with chronic stroke.^12^ No studies have investigated the ability of the ST to accurately assess cardiopulmonary capacity in people with subacute or chronic stroke. Results may be different in people with subacute stroke compared with people with chronic stroke, as the process of motor recovery may confound reproducibility results. Also, no studies have specifically addressed the issue of how soon after a stroke graded exercise testing can be performed safely^2^.

The primary objective of this study was to investigate the feasibility, reproducibility, criterion and convergent validity of the Shuttle Test (ST) to measure cardiopulmonary capacity in people with stroke.

Our secondary objective was to determine if there are differences in the feasibility, reproducibility, criterion and convergent validity of the ST during the subacute versus the chronic phase after stroke.

**Methods**

**Recruitment and subjects**

Stroke survivors receiving rehabilitation were recruited from March – December 2017 from a rehabilitation centre (subacute participants), or from day therapy centres within residential care facilities or private practices specialized in neuro rehabilitation (chronic participants) in the middle of the Netherlands. Subjects were included in the present study if they had a diagnosis of stroke according to the WHO definition ^13^, were aged over 18 years, able to walk with supervision (Functional Ambulation Categories ≥ 3) and be in the final week prior to discharge from the rehabilitation centre. Subacute stroke was defined as time since stroke between 1 and 6 months and chronic stroke was defined as time since onset > 6 months.

Subjects were excluded if they presented with severe cognitive disorder (Mini Mental State Examination <24 points), severe communicative disorder (Utrecht Communication State < 4 points), if they had contraindications to maximal exercise ^14^, such as severe cardiovascular disease or if they were a recurrent faller, defined as  more  than  two  falls in a  six‐month  period ^15^. Falls were defined as “an unexpected event in which the participants come to rest on the ground, floor, or lower level” ^16^.

Data were collected by two experienced exercise physiologists (TB and MV). Both assessors were AED-trained. A physician was always stand-by in the immediate surroundings to act in case of emergency. The study was approved by the Medical Ethical Board of the Utrecht Medical Center. Written informed consent was obtained from each subject. Subjects were treated in accordance to good clinical practice and the declaration of Helsinki ^17^.

**Procedure**

Prior to the first assessment subjects were asked to perform only light physical activity on the day before testing, and not to exercise or smoke for 2 hours before their test visits as it is known this can influence cardiopulmonary capacity test results.

At the first test visit, pre-participation health screening using the AHA/ACSM Health/Fitness Pre-participation Screening Questionnaire^18^ was performed according to guidelines of the American College of Sports Medicine^18^. Following the screening, demographic data were collected and height and weight measured using calibrated measurement instruments. Recurrent falls were defined based on self-report ^19^. We also measured balance (Performance Oriented assessment of Mobility problems in Elderly patients [POMA])^20^ and functional lower extremity strength (MI)^21^.

The 10m ST was conducted according to the protocol by Verschuren et al.^11^ which starts at 2 km/h and increases speed by 0.25 km/h every level (one minute) to a maximum speed of 7.5 km/h (23 stages). In this protocol, the number of shuttles is the primary outcome, which has been found to be reproducible with an ICC_2,1_ = 0.99^11^. Convergent validity was confirmed by high significant correlations (r_p_ ≥.65, p<0.01) between the ST and 6MWT^12^.

Two cones were set to provide a between-cone distance of 10 meters. The ST required participants to walk between the 2 markers delineating the course, at a set incremental speed determined by an auditory signal (beep), which was played by a standard CD/MP3 player. Each stage lasted about 1 minute. Participants were instructed to keep walking as long as possible, not to talk during the test and to wait to start walking until the beep had sounded. To ensure that the subjects were safe during the test, a researcher assisted them by walking behind or next to them. No gait belts or other devices were used for support, but patients were allowed to use their own assistive devices for walking. An emergency button was always within reach of the subjects and the therapist.

During the test, subjects wore a mobile gas analysis system and a portable wireless ECG (Custocor Custo Med, Ottobrunn, Germany). The mobile gas analysis system consisted of a facemask, a transmitting unit (containing different oxygen and carbon dioxide gas analysers), and a receiving unit. The transmitting unit with facemask and tubing was attached to the subjects with a harness, and the receiving unit was connected to a laptop computer located within 500 m of the transmitting unit. Metabolic stress test software (Metasoft, Version 5.4) was used to measure ECG, heart rate, breath-by-breath minute ventilation, oxygen consumption (VO_2_) carbon dioxide production (VCO_2_), and to calculate the Respiratory Exchange Ratio (RER=VCO_2_/VO_2_). The gas analysis system was fully calibrated immediately before each test as recommend by the manufacturer. The validity and reliability of this system is acceptable^22,23^.

The test finished when the subject was limited by dyspnoea or when the subject was unable to maintain the required speed and failed twice to complete a shuttle before the beep sounded. Subjects were asked not to talk during the test, but to use a hand signal to indicate they wanted to stop the test. After the test subjects were asked to walk back and forth slowly for a few minutes as a cool-down period.

Subjects were then asked to rest for 20 minutes prior to engaging in a supramaximal constant work rate validation procedure bout of exercise^24^ to confirm subjects had reached maximal effort and the highest shuttle they could achieve during the test. Supramaximal means a workload above the peak workload attained during a ST. This validation procedure consisted of a constant work rate test of maximal 3 minutes^25^ at one shuttle higher than the highest shuttle reached in the ST

At the second visit, on average 1 week later, height and weight of the subjects was measured, and subjects were asked to perform the ST again.

**Outcome measures**

The primary variable was the number of shuttles walked.

The secondary variables were:

1. VO_2max_ (ml.kg^-1^.min^-1^), defined as a plateau in VO_2_ or a Respiratory Exchange Rate (RER) of ≥1.05 for 50-64 year olds and RER ≥1.0 for those 65 years and older for both males and females as recommended by Edvardsen et al. ^26^ We considered VO_2peak_ as the average of the last 30 sec of the ST.
2. 1^st^VT. determined by two independent raters using the ventilatory equivalents method, and the nadir of fraction of oxygen (P_ET_O2) according to Binder et al. ^27^When the outcomes were uncertain, the V-slope method was used to verify the 1^st^VT. The found 1^st^VT outcome was confirmed when the RER during this period of time was below 1.0. We calculated two-way random Intraclass Correlation Coefficients with absolute agreement for the interrater reliability of 1^st^ VT and found it to be ICC_2,1_=0.91 with a 95% CI .38 -.98.

**Other outcomes**

Data were collected on age, sex, lesion type, side of stroke, side of hemiplegia, height and weight. The lower extremity Motricity Index (leMI) was used to assess functional strength of the lower extremities ^21^. The minimum score is 0 and the maximum score is 100 (normal strength). The Tinetti Performance Oriented Mobility Assessment-Balance (POMA-B) was used for assessing balance^20^. For the POMA-B the minimum score is 0 and the maximum score 16 (no balance problems). For perceived exertion the Borg score was used (range 6-20).

Data analyses

All analyses were performed with the statistical software package SPSS version 24 for Windows (IBM SPSS Statistics 24). All data were checked with tests of normality using Shapiro Wilks statistics. Percentages were calculated for ordinal data. When normally distributed, means and standard deviations (SD) were calculated for descriptive measures. Medians and ranges were reported for non-normally distributed data. We calculated differences between those who were unable to complete the ST (defined as inability to complete the first shuttle in the first test) and those who completed the test using independent samples t-tests or Mann-Whitney U tests as appropriate.

Linear mixed modelling was used to determine whether there were significant differences in number of completed shuttles when correcting for time of testing (test or retest), MI, POMA-B, sex and age as the independent variables. We assumed a random intercept.

Feasibility was assessed by recording complications and/or adverse effects during or after the ST. In addition, we determined if subjects reached maximal effort during the ST by using a supramaximal constant work rate test.

Reproducibility concerns the degree to which repeated measurements in stable persons (test-retest) provide similar answers. In accordance with Terwee et al.^28^ we made a distinction between reliability and agreement.

For reliability we calculated a two way mixed Intraclass Correlation Coefficient (ICC_2,1_) with absolute agreement of interrater reliability for the determination of the 1^st^VT, the absolute and relative VO_2peak_, HR_peak_ and RER_peak,_ distance walked and number of shuttles for the whole sample, chronic survivors and the subacute survivors^29^. For the interpretation of results, single measure ICC values less than 0.5 are indicative of poor reliability, values between 0.5 and 0.75 indicate moderate reliability, values between 0.75 and 0.9 indicate good reliability, and values greater than 0.90 indicate excellent reliability^29^

To determine the level of agreement, Standard Error of Measurement (SEM) and the smallest detectable change (SDC_ind_) were calculated according to the recommendations of Terwee et al.^28^

For cardiopulmonary criterion validity we determined if the ST stresses the cardiopulmonary system sufficiently to attain at least the 1^st^VT, or VO_2max_ (see definition above). We considered the test valid for stressing the cardiopulmonary system if the 1^st^VT could be determined in 80% of subjects during the ST.

For convergent validity we hypothesized that there would be a strong positive correlation (r ≥ 0.75) between the number of shuttles completed and aerobic capacity, measured as VO_2peak_ mL.min^-1^ and VO_2peak_ mL.kg^-1^.min^-1^. Correlations ranging from 0.00 – 0.25 are considered to indicate no relationship, those from 0.25-0.50 a fair degree of relationship, values of 0.50-0.75 a moderate to good and values above 0.75 a good to excellent relationship^29^. In addition, we performed a forward linear regression analyses with the number of shuttles walked as the dependent variable and the Total Motricity Index score and VO_2peak_ mL.kg^-1^.min^-1^ as the independent variables.

Finally, we analysed whether validity and reproducibility of the ST was different between participants in the subacute versus the chronic phase after stroke by using unpaired t-tests and Mann-Whitney U tests as appropriate.

**Results**

**Subjects**

Twenty-six subjects were recruited for the study. No potential participants were excluded on the basis of the pre-participation screening. N=6 (x%) were unable to complete the first shuttle in the first test. Subjects who were unable to complete the first shuttle were on average older (mean (SD) = 73 (7.4) years, p=0.06), had a lower POMA score (range 9-11, p=0.001) and a lower leMI (mean (SD) = 57.33 (23.1), p=0.006). These six subjects were excluded from the retest.

Twenty subjects completed the test twice. Demographic data for the test-retest sample are shown in Table 1.

Table 1 about here.

Subheading needed – eg test retest reliability?

Linear mixed modelling analysis of the whole sample with the number of completed shuttles as the dependent variables adjusting for sex, age, POMA and leMI as the dependent variables showed no significant effect of time of testing (p=0.06). Please see Table 2.

Table 2 about here.

**Feasibility**

There were no adverse events during or after the ST, including dizziness, syncope, fainting, chest pains or (near) falls while performing the ST. Most (n=17 [85%]) of the subjects stopped due to motor control and/or coordination problems that resulted in the inability to walk the required speed. Fifteen percent of participants reported stopping because they were out of breath. Median (range) Borg scores were 15 (7-17) and 16.5 (7-18) at the test and retest, respectively. The test was viable in all settings (private practice, rehabilitation and day care).

Mean (SD) minutes walked in the supramaximal test was 1.22 (0.64) minutes. No participants were able to complete the 3 minute supramaximal test, suggesting they had reached their maximal capacity during the ST.

**Reproducibility.**

There were no statistically significant differences between the test and retest for all measures reported in Table 3.

In Table 3 the results of the reproducibility of the Shuttle Test are presented with the single measure ICC_2,1 agreement_, SEM and SDC measures for the whole sample. VO_2peak_ L.min^-1^, VO_2peak_ mL.kg^-1^.min^-1^, number of shuttles and meters walked had ICCs > .90 and were considered excellent. For the reproducibility measures for the chronic stroke survivors, see S1 Appendix and for the reproducibility measures for the subacute group, see S2 Appendix.

**Validity**

For criterion validity we determined if the ST stresses the cardiopulmonary system sufficiently to attain at least the 1^st^ VT or VO_2max_ in at least 80% of participating subjects. Ten (50%) out of the 20 subjects attained 1^st^VT in the first test. The same ten subjects and an additional two subjects (60%) attained a 1^st^VT in the retest. One subject aged 79 years attained a RER = 1.04 in the test and one subject aged 65 years attained a RER=1.0 in the retest, suggesting that by our criteria they reached VO_2max_.

Two subjects attained the 1^st^VT at the end of both test and retest and three additional subjects attained the 1^st^VT at the end of the retest. Paired t-testing revealed no significant differences between test and retest (1^st^VT VO_2peak_ L.min^-1^; p=.60 and 1^st^VT VO_2peak_ mL.kg^-1^.min^-1^; p=.74).

Participants who did not attain the 1^st^VT in the first test had lower mean (SD) leMI (80.4 (18.43)) than those who did (90.8 (10.87)), but this difference was statistically not significant. In the retest total leMI was 91.42 (10.21) for those who reached a 1^st^VT versus 76.88 (18.89) for those who did not, this difference was statistically significant (p=0.04). Equally, the POMA was somewhat lower in those who did not attain the 1^st^VT, but this difference of less than one point was not statistically significant between the test and retest.

For convergent validity we hypothesized that there would be a good positive correlation (r ≥ 0.75) between number of shuttles walked and both VO_2peak_ L.min^-1^ and VO_2peak_ mL.kg^-1^.min^-1^.

Two sided Pearson correlations between number of shuttles walked and cardiovascular values were significant (p< 0.01) for VO_2peak_ L.min^-1^ r = .72 (test) and r= .78 (retest) and for VO_2peak_ mL.kg^-1^.min^-1^ r= .76 (test) and r=.86 (retest) (see Figure 1.).

Figure 1 about here.

Subheading needed

The total leMI had a significant association (p< 0.01) of r=0.57 and r=0.58 with the number of shuttles walked for the test and retest, respectively. There was no significant association of the number of shuttles with sex, body height or weight.

Forward multivariate analysis with the number shuttles walked at the first test as the dependent variable and leMI and VO_2peak_ ml.kg^-1^.min^-1^ as independent variables showed an adjusted R^2^ of 0.55 in the final model with VO_2peak_ mL.kg^-1^.min^-1^ (p=0.0001).

At the retest, the adjusted R^2^ was 0.76 with leMI (p=0.049) and VO_2peak_ mL.kg^-1^.min^-1^ (p=0.0001) contributing significantly to the model (see Table 4).

Table 4 about here.

**Differences in people with stroke in the subacute versus chronic phase**

Seven in-patient rehabilitation (subacute) subjects, 4 males and 3 females, were included in the sample. Independent t-testing between the subacute sample and the sample with chronic stroke revealed the subacute sample was significantly younger (p=0.005). Their leMI was significantly higher (p=0.006). The number of meters walked did not significantly differ between groups in the test, but was significantly higher in the retest (p=0.05). VO_2peak_ L.min^-1^ did not differ between the groups. In contrast, VO_2peak_ ml.kg^-1^.min^-1^ was significantly higher in both test (p=0.04) and retest (p=0.04) for the subacute sample. For details, see Appendix 3.

**Discussion**

This study provides insight into the cardiopulmonary response to the ST in subacute and chronic stroke survivors. None of the subjects were able to complete the supramaximal test, suggesting they reached maximal exertion in the ST. Therefore, the ST can be considered a symptom limited maximal exercise test for people with stroke. The metabolic demand of the ST varied widely as the primary limiting factor was motor performance for most participants.

The ST is a safe, feasible and reproducible test for measuring symptom limited maximal exercise capacity with good convergent validity for subacute and chronic stroke survivors. Criterion validity, however, indicates it is not a valid test for measuring maximal cardiopulmonary capacity. Eighty-five percent of subjects in the test-retest sample stopped due to motor and/or coordination problems.. By our criteria, only two subjects (5%) achieved a VO_2max_ in the test or retest; both of these participants had minimal motor impairments. A 1^st^VT could only be determined in 50% of subjects during the first test and in 60% during the second test.

For convergent validity we found a good to excellent relationship between the number of shuttles walked and cardiopulmonary capacity. This was confirmed in the multiple regression analysis where VO_2peak_ mL.kg^-1^.min^-1^ made a significant contribution to the number of shuttles walked. Subjects with higher cardiopulmonary capacity were able to walk more shuttles.

Higher leMI also contributed to a higher amount of shuttles walked, but the effect was small. If the leMI increased by 24 points; e.g. from “full movement against gravity, but weaker than the other side” to “normal strength in the lower extremity”, it would result in an increase of 2.2 shuttles, which is smaller than the SDC_ind_.

The reliability of the number of shuttles walked was excellent with a narrow 95% confidence interval (CI).

Reproducibility was consistent with the results reported by van Bloemendaal et al.^12^ They reported an ICC_2,1 agreement_ of .96 for walking distance and in this study an ICC_2,1_ _agreement_ of .97, with a 95% CI of .92-.99 was found. They found a SEM of 109 meters, while we found a SEM of 64.83 meters. In agreement with Bongers et al.^30^ all measures on the retest were slightly higher, which suggests a learning effect. This is in contrast to Bloemendaal et al.^12^ who did not find a learning effect. Nevertheless, a habituation session prior to the ST is recommended.

The finding that those with lower leMI (i.e. less strength) were less likely to reach a 1^st^VT, matches the subjects’ reporting stopping due to motor / coordination problems. This is in contrast to Marzolini et al.^31^ who reported 76% of subjects had a discernible 1^st^VT during a maximal exercise test on a recumbent bicycle, and did not differ significantly from those who did not reach a 1^st^VT by lower limb motor impairment and balance score.

**Study strengths and weaknesses**

This is the first study to demonstrate reproducibility of a field-based cardiopulmonary capacity / fitness test in people with stroke ^5^. However, our sample size was small and n=x (23%) of the initial sample was unable to perform the first level of the ST. Apart from being very slow walkers, they also had significantly less lower extremity motor strength and balance. The ST may therefore not be a useful test for very slow walkers or for people with severe balance problems.

We tested subjects one week apart, which means we cannot be sure if the results on the subacute sample hold when testing with a longer period in between tests. Prior research showed balance, measured by the POMA, has a confounding effect on distance walked in the 6MWT^32^, but we did not find this effect in this study. As we excluded subjects with a history of falls, potentially excluding those with poorer balance, we may have limited this confounding effect. This could also have positively affected the number of shuttles walked. We did not measure the distance walked, but estimated it from the number of shuttles walked. This could have resulted in overly optimistic reproducibility measures for the distance walked.

**Implications**

Walk tests, such as the commonly used 6-minute walk test (6MWT), have been designed and used as surrogate measures of cardiorespiratory capacity in other populations, but associations between distance walked and cardiopulmonary capacity are low to moderate in stroke survivors ^33^. Correlations between the ST and cardiopulmonary capacity were higher (r>0.72) than those reported for the 6MWT (mean r=0.52, 95% CI 0.42– 0.62, ^33^), suggesting the ST may be a better measure for assessing cardiopulmonary capacity than the 6MWT.

In addition, the ST is a maximal symptom limited exercise test and may be useful as a screener for exercise tolerance and the potential adverse effects of exercise..

Further studies should examine the responsiveness and minimally important change in this population to determine if the test can be used as an outcome measure.

**Clinical messages:**

- The ST is a maximal symptom limited exercise test
- The ST is a safe, feasible and reproducible test in subacute and chronic stroke survivors
- The ST has a good to excellent relationship with cardiopulmonary capacity

Acknowledgements: the authors wish to thank the participating physical therapists/ kinetic therapists: Evelien Bruggemann; Jolien Netjes; Maud Eeuwen; Manoek Ticheler; Marloes Everaers; Judith Meijerink; Marielle Wittekamp; Jonneke Kroes and the stroke survivors who participated in this study.

Conflict of interest: none declared.

Funding: this project was funded by SIA RAAK Publiek: RAAK.PUB03.015

ORCID: Harriet Wittink: <https://orcid.org/0000-0001-5592-2879>

**References**

1.	MacKay-Lyons MJ. Aerobic exercise recommendations to optimize best practices in care after stroke. *Stroke* 2011;42:E614–E614.

2.	Billinger SA, Arena R, Bernhardt J, et al. Physical activity and exercise recommendations for stroke survivors: a statement for healthcare professionals from the American Heart Association/American Stroke Association. *Stroke* 2014;45:2532–53.

3.	Eng JJ, Dawson AS, Chu KS. Submaximal exercise in persons with stroke: test-retest reliability and concurrent validity with maximal oxygen consumption. *Arch Phys Med Rehabil* 2004;85:113–8.

4.	Mackay-Lyons MJ, Makrides L. Exercise capacity early after stroke. *Arch Phys Med Rehabil* 2002;83:1697–702.

5.	Wittink H, Verschuren O, Terwee C, et al. Measurement properties of maximal cardiopulmonary exercise tests protocols in persons after stroke: A systematic review. *J Rehabil Med* 2017;49:689–99.

6.	Boyne P, Reisman D, Brian M, et al. Ventilatory threshold may be a more specific measure of aerobic capacity than peak oxygen consumption rate in persons with stroke. *Top Stroke Rehabil* 2017;24:149–57.

7.	Tang A, Sibley KM, Bayley MT, et al. Do functional walk tests reflect cardiorespiratory fitness in sub-acute stroke? *J Neuroeng Rehabil* 2006;3:23.

8.	Eng JJ, Chu KS, Dawson AS, et al. Functional walk tests in individuals with stroke: relation to perceived exertion and myocardial exertion. *Stroke* 2002;33:756–61.

9.	Salbach NM, Brooks D, Romano J, et al. Cardiorespiratory responses during the 6-minute walk and ramp cycle ergometer tests and their relationship to physical activity in stroke. *Neurorehabil Neural Repair* 2014;28:111–9.

10.	Pang MYC, Eng JJ, Dawson AS. Relationship between ambulatory capacity and cardiorespiratory fitness in chronic stroke: influence of stroke-specific impairments. *Chest* 2005;127:495–501.

11.	Verschuren O, Takken T, Ketelaar M, et al. Reliability and validity of data for 2 newly developed shuttle run tests in children with cerebral palsy. *Phys Ther* 2006;86:1107–17.

12.	van Bloemendaal M, Kokkeler AM, van de Port IG. The shuttle walk test: a new approach to functional walking capacity measurements for patients after stroke? *Arch Phys Med Rehabil* 2012;93:163–6.

13.	Stroke--1989. Recommendations on stroke prevention, diagnosis, and therapy. Report of the WHO Task Force on Stroke and other Cerebrovascular Disorders. *Stroke* 1989;20:1407–31.

14.	Riebe D, Franklin BA, Thompson PD, et al. Updating ACSM’s Recommendations for Exercise Preparticipation Health Screening. *Med Sci Sports Exerc* 2015;47:2473–9.

15.	Aly N and Mailk IH. Appoaching geriatric patients: the frequent fallers. *Scottish Univ Med J* 2013;2:20–7.

16.	Lamb SE, Jorstad-Stein EC, Hauer K, et al. Development of a common outcome data set for fall injury prevention trials: the Prevention of Falls Network Europe consensus. *J Am Geriatr Soc* 2005;53:1618–22.

17.	World Medical Association. World Medical Association Declaration of Helsinki: ethical principles for medical research involving human subjects. *JAMA* 2013;310:2191–4.

18.	American College of Sportsmedicine. *ACSM’s guidelines for exercise testing and prescription*. 6th ed. Philadelphia: Lippincott Williams and Wilkins; 2000.

19.	Heidari S, Babor TF, Castro P De, et al. Sex and Gender Equity in Research: rationale for the SAGER guidelines and recommended use. *BMC Res Integr Peer Rev*;1. Available at https://kopernio.com/viewer?doi=10.1186/s41073-016-0007-6&route=6. 2016.

20.	Tinetti ME. Performance-oriented assessment of mobility problems in elderly patients. *J Am Geriatr Soc* 1986;34:119–26.

21.	Demeurisse G, Demol O, Robaye E. [Motor evaluation in vascular hemiplegia]. *Brux Med* 1979;59:87–94.

22.	Vogler AJ, Rice AJ, Gore CJ. Validity and reliability of the Cortex MetaMax3B portable metabolic system. *J Sports Sci* 2010;28:733–42.

23.	Overstreet BS, Bassett DRJ, Crouter SE, et al. Portable open-circuit spirometry systems. *J Sports Med Phys Fitness* 2017;57:227–37.

24.	Nolan PB, Beaven ML, Dalleck L. Comparison of intensities and rest periods for VO2max verification testing procedures. *Int J Sports Med* 2014;35:1024–9.

25.	Rossiter HB, Kowalchuk JM, Whipp BJ. A test to establish maximum O2 uptake despite no plateau in the O2 uptake response to ramp incremental exercise. *J Appl Physiol* 2006;100:764–70.

26.	Edvardsen E, Scient C, Hansen BH, et al. Reference values for cardiorespiratory response and fitness on the treadmill in a 20- to 85-year-old population. *Chest* 2013;144:241–8.

27.	Binder RK, Wonisch M, Corra U, et al. Methodological approach to the first and second lactate threshold in incremental cardiopulmonary exercise testing. *Eur J Cardiovasc Prev Rehabil* 2008;15:726–34.

28.	Terwee CB, Bot SDM, de Boer MR, et al. Quality criteria were proposed for measurement properties of health status questionnaires. *J Clin Epidemiol* 2007;60:34–42.

29.	Portney, L.G., Watkins MP. *Foundations of clinical research: applications to practice.* 2nd ed. Prentice Hall; New Jersey; 2000.

30.	Bongers BC, Rijks EBG, Harsevoort AGJ, et al. 10-m Shuttle Ride Test in Youth With Osteogenesis Imperfecta Who Use Wheelchairs: Feasibility, Reproducibility, and Physiological Responses. *Phys Ther* 2016;96:679–86.

31.	Marzolini S, Oh P, Corbett D, et al. Prescribing Aerobic Exercise Intensity without a Cardiopulmonary Exercise Test Post Stroke: Utility of the Six-Minute Walk Test. *J Stroke Cerebrovasc Dis* 2016;25:2222–31.

32.	Outermans JC, van de Port I, Kwakkel G, et al. The role of postural control in the association between aerobic capacity and walking capacity in chronic stroke: a cross-sectional analysis. *Eur J Phys Rehabil Med*. Epub ahead of print March 2018. DOI: 10.23736/S1973-9087.18.04987-0.

33.	Outermans J, van de Port I, Wittink H, et al. How strongly is aerobic capacity correlated with walking speed and distance after stroke? Systematic review and meta-analysis. *Phys Ther*;95. Epub ahead of print 2015. DOI: 10.2522/ptj.20140081.

34.	Smith AC, Saunders DH, Mead G. Cardiorespiratory fitness after stroke: a systematic review. *Int J Stroke* 2012;7:499–510.

35.	Meyer K, Samek L, Pinchas A, et al. Relationship between ventilatory threshold and onset of ischaemia in ECG during stress testing. *Eur Heart J* 1995;16:623–30.

Table 1: Demographic variables of the test-retest sample (n=20)

|  | Males  (n=9) | Females  (n=11) | Total  (n=20) |
| --- | --- | --- | --- |
| Age (Years, mean(SD)) | 66.50 (12.76) | 56.09 (17.07) | 59.45 (16.15) |
| Height (cm, mean(SD)) | 176.44 (9.63) | 166.27 (9.25) | 170.85 (10.54) |
| Weight (kg, mean(SD)) | 83.00 (19.04) | 74.05 (15.10) | 78.08 (17.13) |
| Duration stroke (months, median (range)) | 6 (2-38) | 55 (5-220) | 29 (2-220) |
| Hemiplegic side (n (%))  Left  Right  Both  None  Missing | 4 (44.4)  3 (33.3)  1 (11.1)  1 (11.1) | 6 (54.5)  3 (27.3)  2 (18.2) | 10 (50)  6 (30.0)  1 (5.0)  1 (5.0)  2 (10.0) |
| Type of CVA (n (%))  Ischaemic  Haemorrhagic  Unknown | 8 (88.9)  1 (11.1) | 6 (54.5)  4 (36.4)  1 (9.1) | 14 (70.0)  5 (25.0)  1 (5.0) |
| FAC (n (%))  FAC 4  FAC 5 | 2 (22.2)  7 (77.8) | 2 (18.2)  9 (81.8) | 4 (20.0)  16 (80.0) |
| Motricity Index lower extremity (median (range)) | 91 (75-100) | 91 (44-100) | 91 (44 – 100) |
| POMA (median (range)) | 15 (13-16) | 16 (13-16) | 16 (13 – 16) |
| Beta-blockers (n (%)) | 2 (22.2) | 1 (9.0) | 3 (15.0) |
| Assistive devices indoors (n (%))  None  Cane  Walker / cane | 8 (89.0)  1 (11.0) | 10 (90.9)  1 (9.9) | 18 (90.0)  1 (5.0)  1 (5.0) |
| Assistive devices outdoors (n (%))  None  Cane  (rolling) walker  Walker/ cane | 7 (77.8)  1 (11.1)  1 (11.1)  0 | 8 (72.7)  1 (9.1)  1 (9.1)  1 (9.1) | 15 (75.0)  2 (10.0)  2 (10.0)  1 (5.0) |

Table 2: results linear mixed modelling for effect of time of testing on number of Shuttles

| Variable | Estimate | | t | Sig. | 95% Confidence Interval | |
| --- | --- | --- | --- | --- | --- | --- |
|  |  |  |  |  | Lower Bound | Upper Bound |
| Intercept | | -17.69 | -1.18 | 0.26 | -49.71 | 14.34 |
| Time of testing | | 0.48 | 1.99 | 0.06 | -0.03 | 0.98 |
| Sex | | 3.44 | 1.90 | 0.08 | -0.41 | 7.29 |
| POMA | | 1.66 | 1.84 | 0.09 | -0.26 | 3.58 |
| leMI | | 0.110 | 1.80 | 0.09 | -0.02 | 0.23 |
| Age | | -0.15 | -2.63 | 0.02 | -0.27 | -0.03 |

Table 3. Reproducibility Shuttle Test whole sample

| Mean (SD) | Test  (n=20) | Retest  (n=20) | Single measure ICC_1,2_ agreement  95% CI | SEM | SDC_ind_ | SDC_group_ |
| --- | --- | --- | --- | --- | --- | --- |
| VO_2peak_ (L.min^_1^) | 1.57 (0.66) | 1.63 (0.70) | 0.93  (0.84; 0.97) | 0.18 | 0.50 | 0.14 |
| VO_2peak_  (mL.kg^-1^.min^_1^) | 20.30 (7.51) | 21.05 (7.67) | 0.90  (0.77; 0.96) | 2.40 | 6.65 | 1.85 |
| HR_peak bpm_ | 124.85 (29.72) | 125.50 (28.57) | 0.89  (0.75; 0.96) | 9.67 | 26.80 | 7.43 |
| RER_peak_ | 0.91 (0.08) | 0.93 (0.07) | 0.69  (0.38; 0.87) | 0.04 | 0.12 | 0.03 |
| distance walked meters | 593.05 (374.28) | 632.50 (374.28) | 0.97  (0.92; 0.99) | 64.83 | 179.69 | 49.84 |
| # of Shuttles | 9.93 (5.30) | 10.40 (5.45) | 0.98  (0.94; 0.99) | 0.76 | 2.11 | 0.58 |
| 1^st^VT (L.min^-1^) | 1.67 (.43) | 1.71 (.53) | 0.84 (0.49; 0.96) | 0.19 | 0.54 | 0.15 |
| 1^st^VT  (mL.kg^-1^.min^-1^) | 20.5 (4.95) | 20.9 (3.87) | 0.67  (0.09; 0.91) | 2.55 | 7.10 | 1.96 |

HR_peak_ = peak Heart Rate, RER_peak_ -= peak Respiratory Exchange Rate, SEM = Standard error of Measurement, SDC= Smallest Detectable Change

Table 4. Coefficients for number of shuttles (dependent variable) at retest

| Variables | | Standardized Coefficients | t | Sig. | 95% Confidence Interval for B | |
| --- | --- | --- | --- | --- | --- | --- |
|  |  | Beta |  |  | Lower Bound | Upper Bound |
| 1 | (Constant) |  | -1.237 | 0.23 | -6.471 | 1.674 |
|  | RelVO_2peak_T2 | 0.855 | 7.006 | 0.001 | 0.426 | 0.790 |
| 2 | (Constant) |  | -2.510 | 0.02 | -15.674 | -1.356 |
|  | RelVO_2peak_T2 | 0.743 | 6.011 | 0.001 | 0.343 | 0.714 |
|  | leMI | 0.262 | 2.115 | 0.05 | 0.001 | 0.182 |

Figure 1. Scatterplot distance walked in meters and VO_2peak_ mL.kg.min^-1^ test and retest

Test

VO2 peak ml.kg-1.min-1

| Distance walked in meters | VO2peak ml.kg.min |
| --- | --- |
| 350 | 15 |
| 130 | 10 |
| 50 | 14 |
| 520 | 18 |
| 250 | 19 |
| 910 | 19 |
| 301 | 15 |
| 910 | 21 |
| 870 | 26 |
| 70 | 14 |
| 870 | 21 |
| 580 | 18 |
| 410 | 13 |
| 310 | 13 |
| 1060 | 22 |
| 180 | 20 |
| 1060 | 38 |
| 1060 | 32 |
| 910 | 23 |
| 1060 | 35 |

Retest

VO2peak mL..kg.min-1

| Distance walked in meters | VO2ml.kg.min-1 |
| --- | --- |
| 290 | 17 |
| 200 | 10 |
| 90 | 12 |
| 520 | 20 |
| 250 | 19 |
| 870 | 23 |
| 350 | 16 |
| 960 | 18 |
| 870 | 23 |
| 150 | 16 |
| 960 | 25 |
| 620 | 20 |
| 370 | 13 |
| 290 | 13 |
| 1060 | 33 |
| 150 | 17 |
| 1060 | 34 |
| 1060 | 30 |
| 1160 | 25 |
| 1370 | 37 |

Appendix 1. Reproducibility Shuttle Test chronic stroke survivors

| Mean (SD) | Test  (n=13) | Retest  (n=13) | Single measure ICC_1,2_ agreement  95% CI | SEM | SDC_ind_ | SDC_group_ |
| --- | --- | --- | --- | --- | --- | --- |
| VO_2peak_ (L.min^_1^) | 1.44 (0.55) | 1.50 (0.57) | 0.93 (0.79; 0.98) | 0.15 | 0.41 | 0.11 |
| VO_2peak_  (mL.kg^-1^.min^_1^) | 17.15 (4.22) | 17.85 (4.49) | 0.86 (0.61;0.95) | 1.63 | 4.52 | 1.25 |
| HR_peak bpm_ | 116.31 (25.91) | 119.08 (23.90) | 0.91 (0.73; 0.97) | 7.48 | 20.72 | 5.75 |
| RER_peak_ | 0.90 (0.09) | 0.91(0.07) | 0.63 (0.14; 0.87) | 0.05 | 0.14 | 0.04 |
| distance walked (meters) | 478.54 (325.04) | 500.00 (321.25) | 0.99 (0.96; 1.00*) | 32.31 | 89.57 | 24.84 |
| # of Shuttles | 8.42 (4.90) | 8.81 (4.59) | 0.98 (0.94; 1.00) | 0.67 | 1.86 | 0.52 |
| 1^st^VT (L.min^-1^) | 1.69 (0.39) | 1.82 (0.49) | 0.74 (0.75; 0.96) | 0.23 | 0.63 | 0.17 |
| 1^st^VT  (mL.kg^-1^.min^-1^) | 18.17 (2.56) | 19.83 (3.37) | 0.36 (-0.41; 0.87) | 2.39 | 6.63 | 1.84 |

HR_peak_ = peak Heart Rate, RER_peak_ -= peak Respiratory Exchange Rate, SEM = Standard error of Measurement, SDC= Smallest Detectable Change *rounded up

| Mean (SD) | Test  ( n=7) | Retest  (n=7) | Single measure ICC_1,2_ agreement  (95% CI) | SEM | SDC_ind_ | SDC_group_ |
| --- | --- | --- | --- | --- | --- | --- |
| VO_2peak_ (L.min^_1^) | 1.81 (0.81) | 1.89 (0.88) | 0.94  (0.70; 0.99) | 0.21 | 0.57 | 0.22 |
| VO_2peak_  (mL.kg^-1^.min^_1^) | 26.14 (9.05) | 27.00 (9.07) | 0.86  (0.39; 0.97) | 3.39 | 9.40 | 3.55 |
| HR_peak bpm_ | 140.71 (31.65) | 137.43 (34.45) | 0.84 (0.39; 0.97) | 13.23 | 36.68 | 13.86 |
| RER_peak_ | 0.94 (0.07) | 0.97 (0.06) | 0.75 (0.16; 0.95) | 0.03 | 0.09 | 0.03 |
| distance walked (meters) | 805.71 (388.75) | 878.57 (464.88) | 0.94 (0.71; 0.99) | 104.93 | 290.87 | 109.94 |
| # of Shuttles | 12.71 (6.01) | 13.36 (6.02) | 0.96 (0.83; 0.99) | 1.20 | 3.34 | 1.26 |
| 1^st^VT (L.min^-1^) | 1.64 (0.54) | 1.43 (0.53) | 0.95 (0.58; 0.99) | 0.12 | 0.33 | 0.13 |
| 1^st^VT  (mL.kg^-1^.min^-1^) | 24.00 (5.94) | 21.00 (4.24) | 0.75 (-.29; .98) | 2.58 | 7.15 | 2.70 |

Appendix 2. Reliability measures for the subacute group

HR_peak_ = peak Heart Rate, RER_peak_ -= peak Respiratory Exchange Rate, SEM = Standard error of Measurement, SDC= Smallest Detectable Change

Appendix 3: differences between persons with stroke in the subacute and chronic phase

|  | Subacute  (n=7) | Chronic  (n=13) | p-value |
| --- | --- | --- | --- |
| Age (Years, mean (sd)) | 46.43 (14.78) | 66.46 (12.30) | **0.005*** |
| Height (cm, mean (sd)) | 170.29 (9.90) | 171.15 (11.25) | 0.87* |
| Weight (kg, mean (sd)) | 69.43 (14.95) | 82.73 (16.90) | 0.10* |
| Duration stroke (months, mean (sd)) | 7.17 (2.04) | 87.77 (83.12) | **0.004*** |
| Motricity Index lower extremity (median (range)) | 100 (89-100) | 85.0 (44-100) | **0.006**** |
| POMA (median (range)) | 16 (14-16) | 16 (13-16) | 0.94** |
| TEST VO_2peak_ (L.min^_1^, mean (sd) | 1.81 (0.81) | 1.44 (0.56) | 0.23* |
| RETEST VO_2peak_ (L.min^_1^, mean (sd)) | 1.89 (0.88) | 1.50 (0.57) | 0.24* |
| TEST VO_2peak_ (ml.kg^-1^.min^_^1, mean (sd)) | 26.14 (9.05) | 17.15 (4.22) | **0.04*** |
| RETEST VO_2peak_ (ml.kg^-1^.min^_1^, mean (sd)) | 27.00 (9.07) | 17.85 (4.49) | **0.04*** |
| VO_2peak_ (ml.kg^-1^.min^_1^, mean (sd))  TEST  RETEST | 26.14 (9.05)  27.00 (9.07) | 17.15 (4.22)  17.85 (4.49) | **0.04***  **0.04*** |
| HR_peak bpm_ (mean (sd))  TEST  RETEST | 140.71 (31.7)  137.43 (34.5) | 116.31 (25.9) 119.08 (23.9) | 0.08*  0.17* |
| RER_peak_ (mean (sd))  TEST  RETEST | 0.94 (0.07)  0.97 (0.06) | 0.90 (0.03)  0.91 (0.07) | 0.25*  0.49* |
| distance walked (meters, (mean (sd)) TEST  RETEST | 805.71 (388.75) 878.57 (464.88) | 478.54 (325.04) 500.00 (321.25) | 0.06*  **0.05*** |
| number of shuttles (mean (sd))  TEST  RETEST | 12.71 (5.18)  13.36 (6.02) | 8.42 (4.90)  8.81 (4.59) | 0.08*  0.07* |
| Borg after test (median (range))  TEST  RETEST | 15 (7-17)  17 (7-18) | 15 (7-17)  16 (8-18) | 0.82**  0.88** |
| Reason for stopping (n (%))  TEST  Out of breath /tired  Motor limitation/ Coordination  RETEST  Out of breath /tired  Motor limitation/ Coordination | 1 (14.3)  6 (85.7)  0  7 (100) | 4 (30.8)  9 (69.2)  5 (41.7)  7 (58.3) | 0.56***  0.23*** |
| VT reached (Yes, n (%))  TEST  RETEST | 4 (57.1)  6 (85.7) | 6 (46.2)  6 (46.2) | 0.64***  0.09*** |
| 1^st^VT (ml.kg^-1^.min^_1^, mean(sd))  TEST  RETEST | 24.0 (5.94)  22.5 (4.51) | 18.17 (2.56)  19.83 (3.37) | 0.06*  0.61* |

*Independent t-test, **Mann-Whitney U test, ***Chi square
