# Supplementary material for: Feasibility, reproducibility and validity of the 10 meter Shuttle Test in mild to moderately impaired people with stroke
Source: PLoS One. 2020 Oct 28;15(10):e0239203. doi: 10.1371/journal.pone.0239203 (PMC7592795; doi:10.1371/journal.pone.0239203)
Supplement: Attachment — Submitted filename: Dear Editor.docx [file pone.0239203.s006.docx]

Dr. C. English

Academic editor Plos One

Dear dr. English,

Many, many thanks for your thorough, detailed and considerate editing of our paper. We have accepted almost all of your suggestions and we:

based each statement in the abstract results on numerical result

explained in the introduction why the 6MWT is not an adequate measure of cardiopulmonary capacity after stroke

changed references to Vancouver style.

- changed “stroke survivors” to “people with stroke” throughout the paper
- changed Shuttle Test (ST) to 10mST throughout the paper.
- changed “subjects” to “participants” throughout the paper
- changed “shuttles walked” to “shuttles completed” throughout the paper
- maintained one decimal point for SD and for all data except age, height and percentages.
- kept both VO_2peak_ mL.min^-1^ and VO_2peak_ mL.kg^-1^.min^-1^ as the first represents an absolute measure and the second a relative measure.
- gave examples of complications for the feasibility aspect of the study.
- described heart rate was measured with a portable wireless ECG (methods)
- sacrificed the gender differences in Table 1 for separate columns for the subacute, chronic and whole samples.
- Gave a reference for the V-slope method
- deleted all yellow highlights we used for the reviewers in the last round.

We believe your edits have greatly improved the readability of the paper and sincerely hope you agree.

On behalf of all authors,

Sincerely,

Dr. Harriet Wittink
