# Supplementary material for: Feasibility, reproducibility and validity of the 10 meter Shuttle Test in mild to moderately impaired people with stroke
Source: PLoS One. 2020 Oct 28;15(10):e0239203. doi: 10.1371/journal.pone.0239203 (PMC7592795; doi:10.1371/journal.pone.0239203)
Supplement: Attachment — Submitted filename: Response to reviewers_R.docx [file pone.0239203.s007.docx]

Dr. C. English

Academic editor Plos One

Dear dr. English,

Many, many thanks again for your additional thorough, detailed and considerate editing of our paper. We have accepted all of your deletes and we:

based each statement in the abstract results on numerical result

changed all p<0.0001 to p<0.001

replaced “very slow walkers” with people who walk slower than 2 km/h or 0.56 m/s.

replaced all “leMI” with “lower extremity Motricity Index”

We use ICC_2,1 agreement_ consistently throughout the text. You asked whether _agreement_ needs to be in subscript. Although we did not change it, we would be happy to drop _agreement_ from the ICC_2,1_ as we stated in the methods section which ICC we use. We just left it in to remind the reader.

In the results section, we now report mean differences with their 95% confidence intervals wherever applicable

In table 3, we took out decimal points for HR beats per second.

We made some minor changes to the Tables to ensure consistent wording.

We sincerely hope you are now content with the paper as it stands,

On behalf of all authors,

Sincerely,

Dr. Harriët Wittink
